# Developing long bones respond to surrounding tissues by *trans*-pairing of periosteal osteoclasts and endocortical osteoblasts

**DOI:** 10.1242/dev.202194

**Published:** 2024-09-05

**Authors:** Yukiko Kuroda, Masaki Yoda, Katsuhiro Kawaai, Motoharu Tatenuma, Toshihide Mizoguchi, Shinichirou Ito, Masataka Kasahara, Yanlin Wu, Hidekazu Takano, Atsushi Momose, Koichi Matsuo

**Affiliations:** ^1^ Laboratory of Cell and Tissue Biology, Keio University School of Medicine, 35 Shinanomachi, Shinjuku, Tokyo 160-8582, Japan; ^2^ Oral Health Science Center, Tokyo Dental College, Tokyo 101-0061, Japan; ^3^ Department of Pharmacology, Tokyo Dental College, Tokyo 101-0061, Japan; ^4^ Institute of Multidisciplinary Research for Advanced Materials (IMRAM), Tohoku University, Katahira 2-1-1, Aoba, Sendai Miyagi 980-8577, Japan; ^5^ JASRI/SPring-8, 1-1-1 Kouto, Sayo-cho, Hyogo 679-5198, Japan

**Keywords:** Osteoclasts, Osteoblasts, Modeling drift, Cortical canals, Trans-cortical vessels, Sciatic nerve transection

## Abstract

Developing long bones alter their shape while maintaining uniform cortical thickness via coordinated activity of bone-forming osteoblasts and bone-resorbing osteoclasts at periosteal and endosteal surfaces, a process we designate *trans*-pairing. Two types of *trans*-pairing shift cortical bone in opposite orientations: peri-forming *trans*-pairing (peri-*t*-p) increases bone marrow space and endo-forming *trans*-pairing (endo-*t*-p) decreases it, via paired activity of bone resorption and formation across the cortex. Here, we focused on endo-*t*-p in growing bones. Analysis of endo-*t*-p activity in the cortex of mouse fibulae revealed osteoclasts under the periosteum compressed by muscles, and expression of RANKL in periosteal cells of the cambium layer. Furthermore, mature osteoblasts were localized on the endosteum, while preosteoblasts were at the periosteum and within cortical canals. X-ray tomographic microscopy revealed the presence of cortical canals more closely associated with endo- than with peri-*t*-p. Sciatic nerve transection followed by muscle atrophy and unloading induced circumferential endo-*t*-p with concomitant spread of cortical canals. Such canals likely supply the endosteum with preosteoblasts from the periosteum under endo-*t*-p, allowing bone shape to change in response to mechanical stress or nerve injury.

## Teaser

**Highlighted Article:** Triggering of *trans*-pairing by periosteal osteoclasts leads to endocortical bone formation in association with cortical canals in growing long bones.

## INTRODUCTION

The major functions of the skeleton are to protect encased organs, support the body and facilitate movement in the presence of force and gravity. Once a shell of cortical bone is formed, its external shape is modified through bone modeling during growth periods as a functional adaptation to mechanical stress (Frost, 2004), resulting in grooves, fossae, crests, ridges and other structures on cylindrical primitive long bones. Bone formation or resorption during modeling occurs on a given surface, while bone formation during remodeling occurs following bone resorption to replace old or damaged matrix (Allen and Burr, 2019). Displacement of the bone shaft also occurs by modeling drift (also known as osseous drift or cortical drift), a process consisting of periosteal bone formation on the leading edge and resorption on the lagging edge of the cortex (Enlow, 1962; Maggiano et al., 2015, 2016). Once morphologically established, cortical bone is maintained by bone remodeling through the activity of basic multicellular units (BMUs) and, in large animals, osteons (Haversian systems).

Use of dynamic axial loading models in mice focusing on the ulna (Robling and Turner, 2002), tibia (Birkhold et al., 2017; Carriero et al., 2018; Pereira et al., 2015) or fibula (Moustafa et al., 2009) has allowed analysis of osteogenic responses to mechanical loading at the periosteum and endosteum of long bones. The osteogenic response to loading is promoted by reduced expression of *Sost* (which encodes the Wnt signaling antagonist sclerostin) in osteocytes (Tu et al., 2012). Micro-CT-based finite element analysis can predict strain and appositional growth in bone (Thiagarajan et al., 2014; van Rietbergen et al., 1995). Generally, in long bones, outward cortical appositional growth results from periosteal bone formation and endocortical bone resorption (Enlow, 1965). Such outward growth of cortical bone is inducible by both tensile and compressive strain with axial loading. Curiously, however, periosteal resorption cannot be induced in axial loading experiments (Birkhold et al., 2016).

Periosteal bone resorption generally occurs in response to static cross-sectional compression perpendicular to the periosteal surface; thus, growing bones can flexibly modify their external shape to accommodate neighboring structures such as muscle, brain, lungs or teeth. We have previously reported that cortical layers of mouse basioccipital bone in the cranial base are resorbed by osteoclasts on the compression side (the endocranial concave surface) facing the enlarging brain as it applies mechanical stress, whereas on the tensile side (ectocranial convex surface), bone is formed by osteoblasts (Edamoto et al., 2019). In this context, reciprocal localization of osteoclasts and osteoblasts across cortical bone is referred to as ‘*trans*-pairing’. *Trans*-pairing-driven bone drift occurs in an osteoclast-dependent manner based on analysis of osteopetrotic mice and mice injected with anti-RANKL antibody or osteoprotegerin (Edamoto et al., 2019; Piemontese et al., 2017).

In long bones and ribs, movement of cortical bone is induced by periosteal resorption paired with endocortical deposition (Enlow, 1965; Galea et al., 2021). Follistatin-induced muscle hypertrophy enhances movement of cortical bone in young adult mice by inducing osteoclast resorption at the periosteum and osteoblast activation on the opposing side (Chan et al., 2021). In orthodontic force-mediated tooth movement, the bone resorption surface emerges at the compression side of the alveolar bone, whereas bone formation occurs on the opposite tension side (Seki et al., 2023). Abundant expression of *Tnfsf11* (which encodes the osteoclastogenic cytokine RANKL) in osteocytes is crucial for alveolar bone resorption on the surface undergoing compressive stress (Shoji-Matsunaga et al., 2017). However, how the location or direction of *trans*-pairing is regulated in long bones remains obscure.

Lightsheet fluorescence microscopy combined with tissue-clearing methods has been used to analyze cortical structure/surfaces of murine long bones (Greenbaum et al., 2017; Tainaka et al., 2018). Phase-contrast X-ray tomographic microscopy can also reveal 3D structures in bone and soft tissue at the single-cell level (Kuroda et al., 2021; Matsuo et al., 2015, 2021; Nango et al., 2016). In microscopy, there is a trade-off between resolution and field of view: higher resolution comes at the expense of a narrower field of view. Thus, in this study, we chose fibula, the thinnest of limb bones (including humerus, radius, ulna, femur, tibia and fibula), for analysis in order to increase resolution. Using these 3D microscopy methods combined with histological techniques, we analyzed cortical structures of the fibula in developing mice. Our data suggest that cortical canals provide the structural basis of endo-forming *trans*-pairing (endo-*t*-p), allowing movement of cortical bone without loss of mechanical strength. We propose that endo-*t*-p of periosteal osteoclasts with endocortical osteoblasts is a major cellular mechanism that shapes growing bone.

## RESULTS

### Pairing of bone resorption and formation across the fibular cortex

**Fig. 1.**
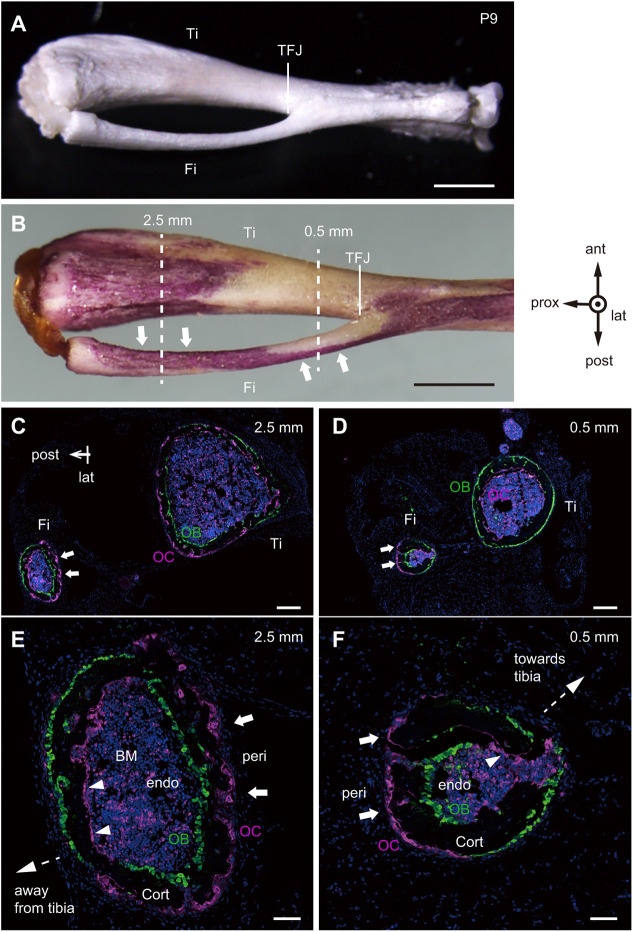
**Trans-pairing across the fibular cortex in P9 mice.** (A) Lateral view of the right tibia (Ti) and fibula (Fi) from a P9 female mouse. TFJ, tibia-fibula junction. Scale bar: 1 mm. (B) Whole-mount TRAP staining of a lower hindlimb of a female P9 mouse. Dotted lines at 2.5 mm and 0.5 mm from the TFJ indicate cross-section levels assessed in C-F. Arrows indicate TRAP-positive periosteal surfaces. Scale bar: 1 mm. lat, lateral; prox, proximal; ant, anterior; post, posterior. (C-F) Immunofluorescence detection of osteoclasts (magenta; MMP9 positive) and osteoblasts (green; osteocalcin positive) in frozen cross-sections of undecalcified tibia and fibula at the 2.5 mm (C,E) and 0.5 mm (D,F) levels isolated from P9 mice (four female). DAPI nuclear stain is in blue. Scale bars: 200 µm in C,D; 50 µm in E,F (higher magnifications of C,D). BM, bone marrow; Cort, cortical bone; peri, periosteum; endo, endosteum; OB, osteoblast; OC, osteoclast. Arrows indicate periosteal resorption surfaces. Arrowheads indicate endocortical resorption surfaces.

Developmentally, cylindrical cartilage templates become cylindrical bones via endochondral ossification. Once cortical bone is established, two parallel long bones in the lower hindlimb are gradually transformed by osteoclasts and osteoblasts into tibia (thicker) and fibula (thinner) (Sharir et al., 2011). The tibia (anterior) and fibula (posterior) become Y-shaped by fusing from the mid-diaphysis to the distal end, as seen in P9 mice (Fig. 1A). To analyze bone resorption, we assessed bone resorption areas on the periosteal surface of the tibia and fibula in P9 mice using whole-mount activity staining for TRAP, a marker of osteoclastic bone resorption. In the proximal fibula at 2.5 mm proximal (towards the knee) to the tibia-fibula junction (TFJ), we observed TRAP-positive areas on the anterior surface facing the tibia (Fig. 1B, downward arrows). In the more distal fibula at 0.5 mm to the TFJ, we observed TRAP-positive areas on the posterior surface facing away from the tibia (Fig. 1B, upward arrows).

We next visualized matrix metalloproteinase 9 (MMP9)-positive osteoclasts (magenta) and osteocalcin-positive osteoblasts (green) in fibula cross-sections from P9 mice at 2.5 and 0.5 mm proximal to the TFJ (Fig. 1C,D). Similar to ‘*trans*-pairing’ seen in the cranial base (Edamoto et al., 2019), bone formation and resorption surfaces occurred in a pairwise manner across cortical bone. At the 2.5 mm level (Fig. 1E), osteoclasts were localized to the periosteal (arrows) and endocortical (arrowheads) surfaces that both face the tibia. As osteoblasts were aligned pairwise across cortical surfaces, such cellular distribution resulted in posterior drift of the fibula at 2.5 mm, moving it away from the tibia. By contrast, at the 0.5 mm level (Fig. 1F), the orientation of paired osteoclast-osteoblast surfaces was reversed, resulting in anterior drift and movement of the fibula towards the tibia. Histological analysis using TRAP-tdTomato mice (Kikuta et al., 2013), which express a red fluorescent marker in osteoclasts, also revealed periosteal osteoclasts localized at the anterior and posterior periosteal surfaces at 2.5 mm and at 0.5 mm, respectively, from the TFJ (Fig. S1A,B, magenta). *Trans*-pairing was observed in the developing fibula with modeling drift at P9, P16, P23 and P30 (Fig. S2). These results suggest that ‘*trans*-pairing’ of bone resorption and formation surfaces across cortical bone induces bone drift in two opposite orientations: peri-*t*-p increases bone marrow space and endo-*t*-p decreases that space through paired bone resorption and formation activities across cortical bone to establish adult bone morphology.

### Sites of periosteal bone resorption correspond to muscle compression

**Fig. 2.**
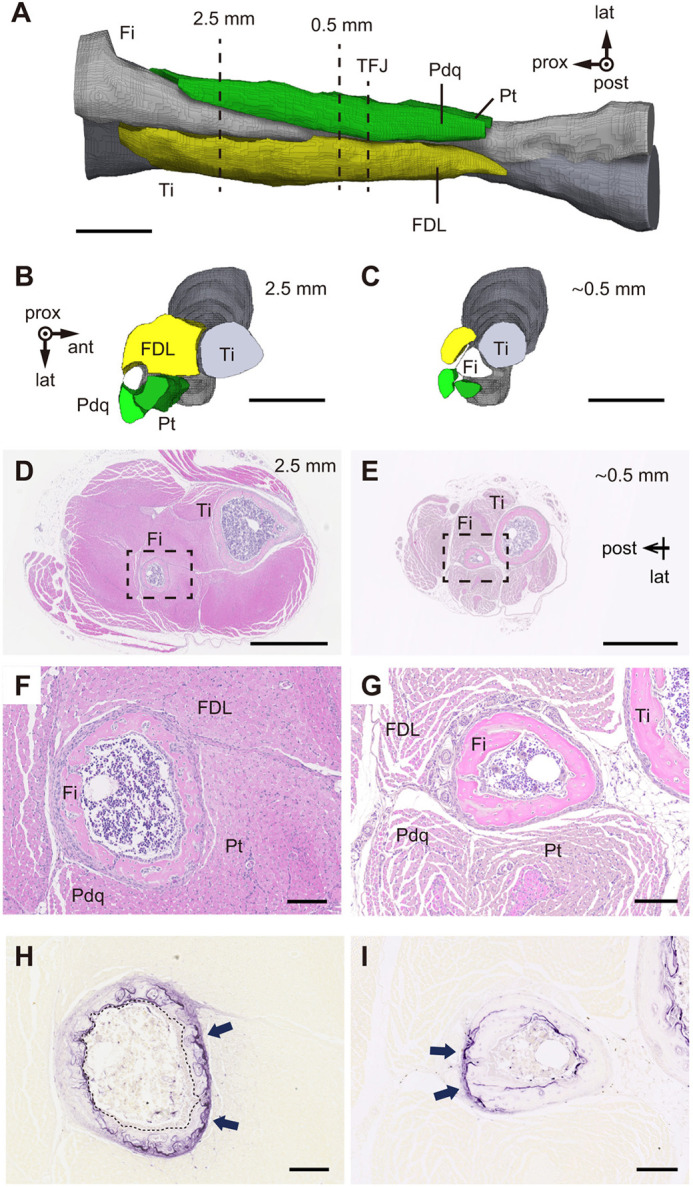
**Periosteal bone resorption occurs at areas corresponding to muscle compression.** (A) Micro-CT image of right lower hindlimb of a P9 male mouse, created through digital segmentation of I_2_KI-enhanced muscles surrounding the fibula (Fi). FDL, flexor digitorum/hallucis longus; Pt, peroneus tertius; Pdq, peroneus digiti quarti; Ti, tibia. Scale bar: 1 mm. (B,C) Cross-sectional views at 2.5 mm (B) and 0.5 mm (C) proximal to the tibia-fibula junction (TFJ) of A. Scale bars: 1 mm. (D,E) Hematoxylin and Eosin staining of cross-sections at 2.5 mm (D) and 0.5 mm (E) from the TFJ in a P9 male mouse. Scale bars: 1 mm. (F) Higher magnification of area outlined in D. Scale bar: 100 µm. (G) Higher magnification of area outlined in E. Scale bar: 100 µm. (H,I) TRAP activity staining of sections adjacent to F and G, respectively. Scale bars: 100 µm. Arrows indicate TRAP-positive periosteal surfaces.

**Fig. 3.**
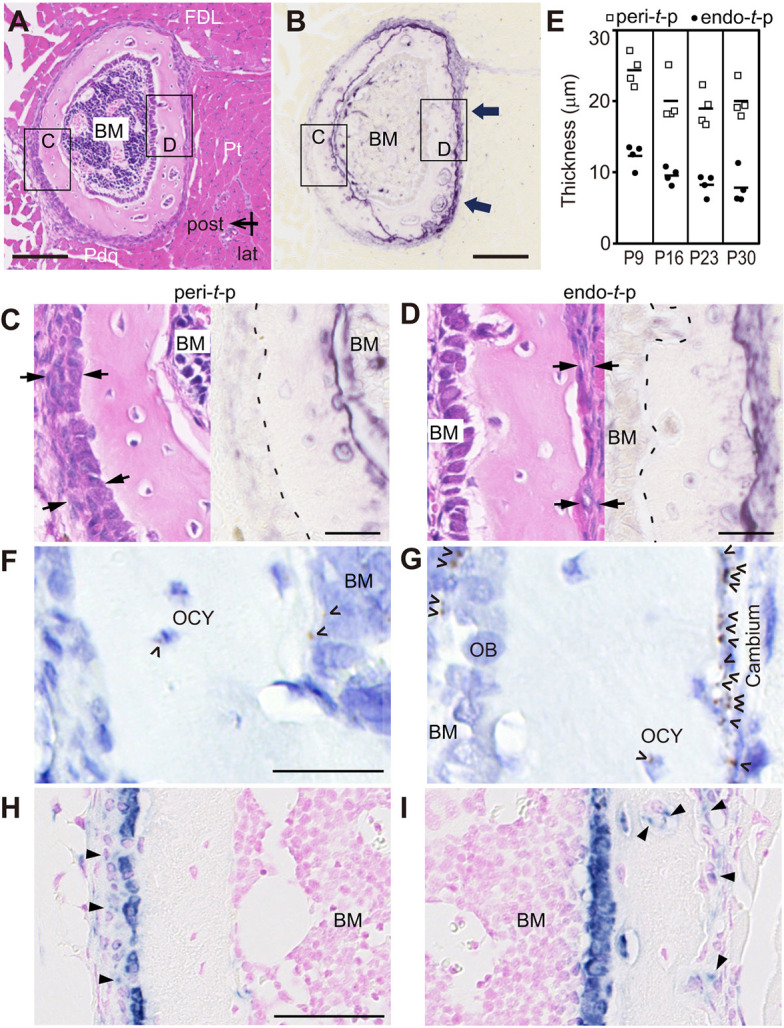
***Tnfsf11* (RANKL) is expressed in cambium cells of the fibular periosteum covered by muscle.** (A) Hematoxylin and Eosin staining of fibula cross-sections at 2.5 mm from the tibia-fibula junction (TFJ) in a P16 male mouse. FDL, flexor digitorum/hallucis longus; Pt, peroneus tertius; Pdq, peroneus digiti quarti; BM, bone marrow. Scale bar: 100 µm. (B) TRAP activity staining of sections adjacent to A. Arrows indicate robust TRAP activity. Scale bar: 100 µm. (C,D) Magnified view of peri-*t*-p (C) and endo-*t*-p (D) regions in A and B. Periosteal thickness is indicated by arrows. Dashed line indicates the periosteal surface in C and the endocortical surface in D. Scale bars: 25 µm. (E) Periosteal thickness at peri- and endo-*t*-p fibular cortices from P9, P16, P23 and P30 male mice (*n*=4 slices for each age of mouse). Horizontal lines indicate mean value of each group. (F,G) *In situ* hybridization of *Tnfsf11* in peri-*t*-p (F) and endo-*t*-p (G) cortices of fibula at 2.5 mm from the TFJ in a P16 mouse (two male) indicates *Tnfsf11* positivity. OCY, osteocyte. Scale bar: 25 µm. (H,I) *In situ* hybridization of *Col1a1* in peri-*t*-p (H) and endo-*t*-p (I) cortices of fibula at 3.0 mm from TFJ in a P16 mouse (two male). Scale bar: 50 µm. Arrowheads indicate preosteoblasts weakly positive for *Col1a1* detected at the periosteum of peri-*t*-p cortex, as well as at the periosteum and in cortical canals of endo-*t*-p cortex.

To identify mechanisms underlying resorption of the periosteal bone surface, we examined tissues surrounding the P9 fibula. Micro-CT imaging revealed that three muscles – the flexor digitorum/hallucis longus (FDL), peroneus tertius (Pt) and peroneus digiti quarti (Pdq) – were in contact with the fibular periosteum (Charles et al., 2016) (Fig. 2A). Notably, the FDL and Pt were located anterior to the fibula (between the fibula and tibia) at 2.5 mm proximal to the TFJ (Fig. 2B), whereas at ∼0.5 mm proximal to the TFJ, FDL, Pt and Pdq muscles contacting the fibula were located posterior to the fibula (Fig. 2C). We next examined histological cross-sections of the lower hindlimb in P9 mice (Fig. 2D,E). In agreement with micro-CT analysis, the anterior (tibial side) of the fibula was surrounded by the FDL, Pt and Pdq at 2.5 mm (Fig. 2F), but that side was free of muscles at 0.5 mm from the TFJ (Fig. 2G). Consistent with areas surrounded by muscles, intense TRAP activity was localized to the anterior and posterior periosteum at 2.5 and 0.5 mm from the TFJ, respectively, at P9 (Fig. 2H,I). At P16, similar strong TRAP activity at the periosteum in contact with muscles was also detected at 2.5 mm from the TFJ (Fig. 3A,B). To confirm whether muscle volumetric expansion compresses the periosteum, we compared thickness of the periosteum at the peri- and endo-*t*-p cortices (Fig. 3C, peri-*t*-p; Fig. 3D, endo-*t*-p) on histological sections. The periosteum was thinner at the endo-*t*-p than the peri-*t*-p cortex in developing fibulae (Fig. 3E). These data suggest that the periosteum of the endo-*t*-p cortex is more compressed than that of peri-*t*-p cortex, and that osteoclastic bone resorption surfaces are established in response to muscle compression, initiating *trans*-pairing of periosteal osteoclasts and endocortical osteoblasts.

To test whether non-physiological mechanical compression induces osteoclastic bone resorption, we implanted a small hex nut on the bone-forming external surface of calvaria under the skin to apply force directly to the growing bone. TRAP-positive areas were ectopically induced on the external surface of the calvaria where the nut was placed (Fig. S3). The data suggest that mechanical compression applied perpendicular to the cortical surface may induce bone resorption. Nevertheless, the potential for inflammation-induced bone resorption cannot be ruled out.

### The periosteum under muscles induces osteoclastogenesis and preosteoblast activation

To identify cells that express *Tnfsf11* (which encodes osteoclastogenic RANKL) in response to muscle compression at the periosteum, we performed *in situ* hybridization using fibular cross-sections from P16 mice taken 2.5 mm proximal to the TFJ. To increase detection sensitivity, we employed RNAscope *in situ* hybridization technology, which allows visualization of each *Tnfsf11* mRNA molecule as a punctate brown dot under standard bright-field microscopy (Fig. 3F,G). We observed more-robust *Tnfsf11* expression in cells in the cambium layer of the endo-*t*-p periosteum (Fig. 3G) compared with peri-*t*-p periosteum, osteocytes and bone marrow (Fig. 3F,G). These data suggest that the primary cells responding to muscle compression are cells in the inner layer of the endo-*t*-p periosteum.

We next used *in situ* hybridization with a *Col1a1* probe to detect osteoblast-lineage cells. As seen in our analysis of osteocalcin-positive osteoblasts (Fig. 1E), we detected osteoblasts highly positive for *Col1a1* at the periosteum of the peri-*t*-p cortex (Fig. 3H) and at the endosteum of endo-*t*-p cortical bone (Fig. 3I). In the peri-*t*-p cortex, preosteoblasts weakly positive for *Col1a1* were present above osteoblasts at the periosteum (Fig. 3H, arrowheads). Interestingly, in the endo-*t*-p cortex, we detected preosteoblasts within both periosteum and cortical canals (Fig. 3I, arrowheads). These data support the hypothesis that in the endo-*t*-p cortex, preosteoblasts induced at the periosteal osteoclast surface likely travel through cortical canals to the endosteum.

### Dense cortical canals define endo-*t*-p

**Fig. 4.**
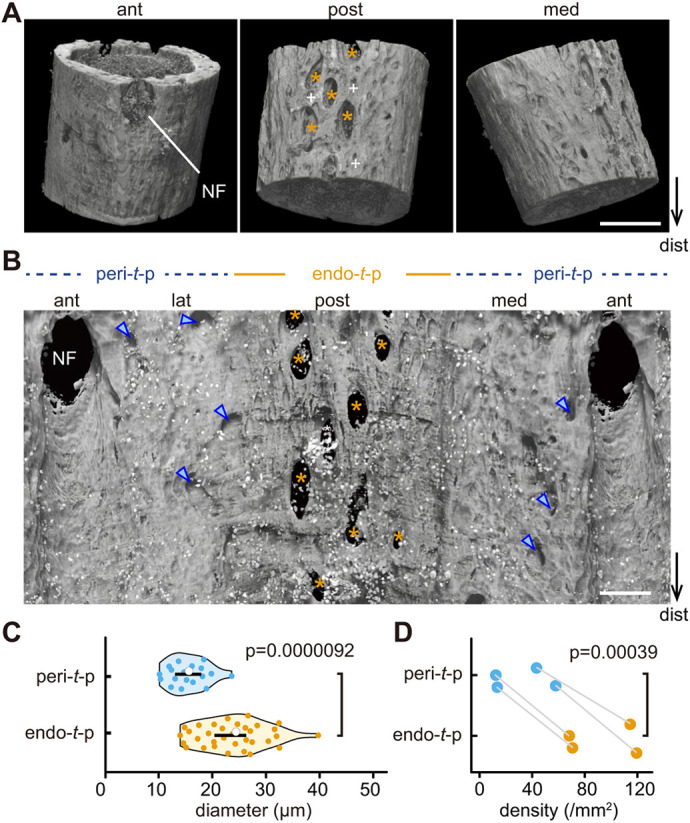
**Phase-contrast X-ray tomographic microscopy of peri- and endo-*t*-p cortices.** (A) 3D morphology of right fibula 0.5-1 mm proximal to the tibia-fibula junction (TFJ) of a P9 male mouse. Scale bar: 100 µm. Plus signs indicate exposed osteocyte lacunae. Asterisks indicate canals across cortical bone. ant, anterior; post, posterior; med, medial; dist, distal; NF, nutrient foramen. (B) Cylindrical panoramic image including duplicated anterior regions of the endocortical surface of the fibula shown in A. Asterisks and arrowheads indicate cortical canals in endo-*t*-p and peri-*t*-p cortices, respectively. (C) Analysis of canal diameter within peri- and endo-*t*-p cortices of fibulae (*n*=4, female and male mice) at 0.5 to 1 mm above the TFJ. Statistical analysis was performed using a Mann–Whitney *U* test. (D) Cortical canal density in peri-*t*-p or endo-*t*-p cortex of fibula analyzed in C. Statistical analysis was performed using a paired *t*-test.

To identify structures linking the periosteal bone resorption surface to the endocortical bone formation surface across cortical bone, we focused on regions of cortical bone of the P9 fibula undergoing endo-*t*-p where periosteal osteoclasts and endocortical osteoblasts were seen in histological cross-sections (Fig. 1). For this analysis, we visualized bone 3D structure using phase-contrast X-ray tomographic microscopy at high resolution (< 0.2 µm voxel size). Interestingly, the posterior cortical bone of the fibula at 0.5-1 mm above the TFJ contained numerous canals across the bone (Fig. 4A, middle panel, asterisks) as well as osteocyte lacunae likely resulting from periosteal bone resorption (Fig. 4A, middle panel, plus signs), suggesting that cortical canals are characteristic of endo-*t*-p.

When we generated a cylindrical panoramic image of the endocortical surface of the fibula *in silico*, that analysis visualized trans-cortical canals in the endo-*t*-p cortex of the posterior fibula (Fig. 4B, asterisks in endo-*t*-p) and in the peri-*t*-p cortex of the medial-anterior-lateral fibula (Fig. 4B, arrowheads in peri-*t*-p). Quantitative analysis, which excluded the large nutrient foramen, indicated that both the diameter (∼15 µm) and density (< 60/mm^2^) of cortical canals in the peri-*t*-p cortex were smaller than the diameter (∼24 µm) and density (> 60/mm^2^) of comparable structures in the endo-*t*-p cortex (Fig. 4C,D). Therefore, the existence of canals in cortical bone is primarily associated with endo- rather than peri-*t*-p.

### Cortical canals contain capillary vessels, osteoclasts and osteoblasts

**Fig. 5.**
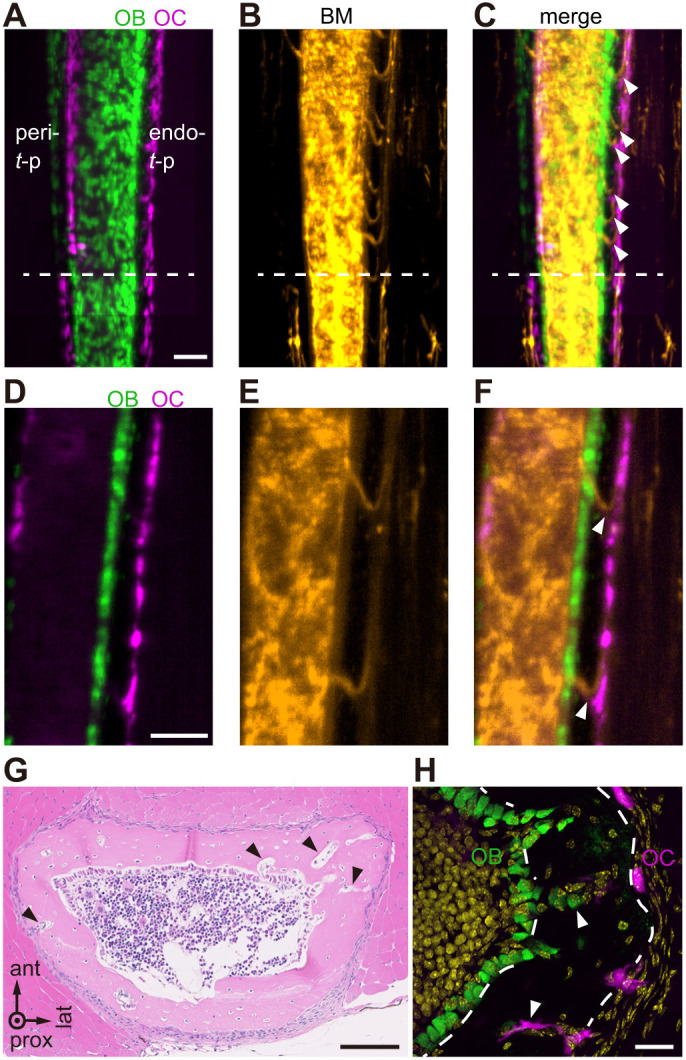
**Cortical canals contain capillary vessels, osteoclasts and osteoblasts.** (A-C) Lightsheet fluorescence microscopy detection of osteoblasts (OB, green; *Col1a1*-AcGFP positive), osteoclasts (OC, magenta; TRAP-tdTomato positive) and the vasculature (orange; tomato lectin positive) in P16 fibula [at ∼1.5 mm above the tibia-fibula junction (TFJ)] of TRAP-tdTomato:*Col1a1*-AcGFP mice (male and female) injected with tomato lectin. 100 µm projection images are shown. Both peri- and endo-*t*-p are detectable above dashed line. Arrowheads indicate trans-cortical vessels. BM, bone marrow. Scale bar: 100 µm. (D-F) Magnified 10 µm projection images taken at the endo-*t*-p cortex. Scale bar: 100 µm. (G) Hematoxylin and Eosin staining of cross-sections of fibula 4 mm above the TFJ in a P30 male mouse. Arrowheads indicate cortical canals. Scale bar: 100 µm. (H) Cellular localization of osteoblasts (green, osteocalcin-positive) and osteoclasts (magenta, TRAP-tdTomato-positive) in cortical bone of fibula 4 mm above the TFJ in a P28 male mouse. DAPI nuclear stain is in yellow. Arrowheads indicate osteoclasts (magenta) or osteoblasts (green) within canals. Dashed lines indicate edges of cortical bone. Scale bar: 20 µm.

To determine whether canals in cortical bone contain a network of capillary vessels connecting periosteum to endosteum, we injected fluorescent tomato lectin into the retro-orbital plexus of TRAP-tdTomato:*Col1a1*-AcGFP double-transgenic mice at P16, which exhibit osteoclasts marked by red fluorescence and osteoblasts marked by green. Trans-cortical vessels are capillaries that traverse the entire cortical thickness by connecting bone marrow with the periosteal circulation (Asghar et al., 2020; Grüneboom et al., 2019). At cortical bone, where we had detected endo-*t*-p (Fig. 5A, endo-*t*-p), we observed multiple trans-cortical vessels (Fig. 5B,C). By contrast, in cortical bone exhibiting peri-*t*-p (Fig. 5A, peri-*t*-p), we observed few trans-cortical vessels (Fig. 5B,C). Moreover, analysis at higher magnification revealed several trans-cortical vessels connecting the periosteal osteoclast surface to the marrow space (Fig. 5D-F). Histological analysis also detected multiple cortical canals in the endo-*t*-p region (Fig. 5G, arrowheads), and immunostaining revealed both osteoblasts and osteoclasts in those canals (Fig. 5H). These data demonstrate that the endo-*t*-p cortex contains more abundant and larger transverse canals than does the peri-*t*-p cortex, and that capillary vessels, osteoblasts and osteoclasts are present in these canals.

### Sciatic nerve transection induces circumferential endo-*t*-p

**Fig. 6.**
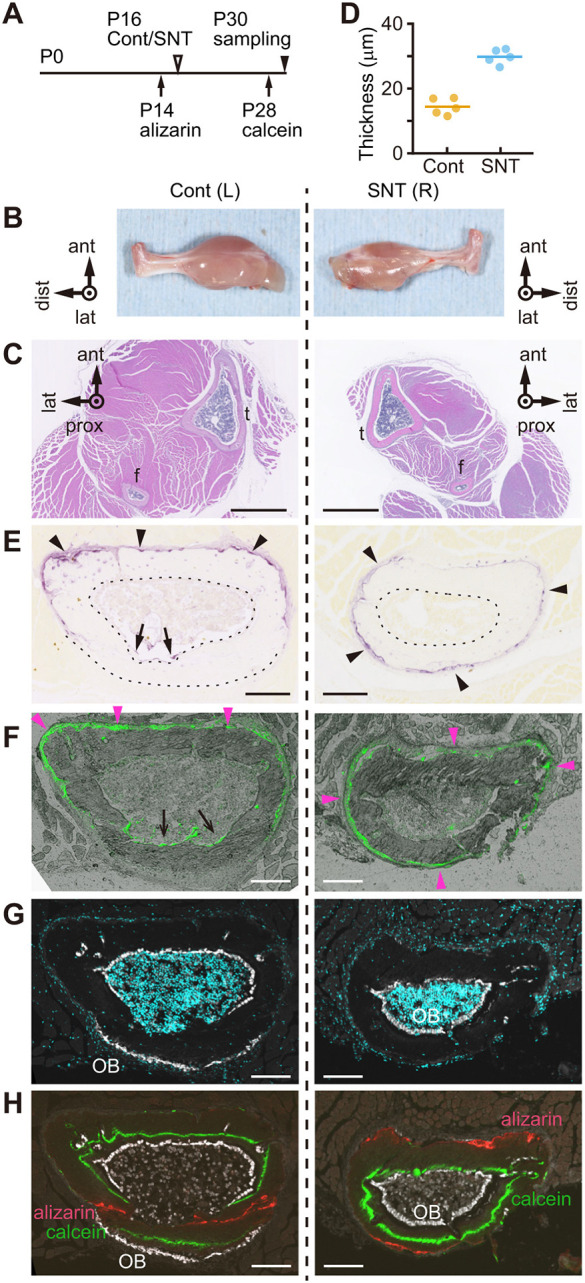
**Sciatic nerve transection (SNT) abolishes directional pairing of bone resorption and formation.** (A) Schematic showing timeline of SNT. (B) Control (Cont) sham-operated left (L) hindlimb (left); right (R) hindlimb (right) subjected to SNT at P16. Both were analyzed 2 weeks later. There is muscle atrophy of the SNT hindlimb (*n*=5 mice, two female and three male). lat, lateral; dist, distal; ant, anterior. (C-G) Histological analyses of SNT and control hindlimbs described above. (C-E) Paraffin-embedded cross-sections at 3.5 mm from the tibia-fibula junction (TFJ). (C) Hematoxylin and Eosin staining of fibula (f) and tibia (t) of a male mouse. Scale bars: 1 mm. (D) Periosteal thickness at anterior cortices of Cont (*n*=2) and SNT fibulae (*n*=3) from male and female mice. A total of five paraffin-embedded sections were measured for each group. Horizontal lines indicate mean value of each group. (E) TRAP activity staining. Arrowheads and arrows indicate periosteal and endocortical bone resorption surfaces, respectively. Scale bars: 100 µm. (F-H) Frozen sections of fibula of a female mouse 4.5 mm from the TFJ. (F) TRAP activity staining using ELF97 as substrate (green). Arrowheads and arrows indicate periosteal and endocortical bone resorption surfaces, respectively. Scale bars: 100 µm. (G) Osteocalcin immunostaining (white) and DAPI staining (cyan). Scale bars: 100 µm. (H) Labeling of bone with alizarin complexone (red) and calcein (green). Also shown is osteocalcin immunostaining (white). Scale bars: 100 µm. G is the same frozen section as H without showing alizarin and calcein labeling.

The sciatic nerve innervates most muscles of the lower limb as well as bone, especially in the periosteum (Chartier et al., 2018; Tomlinson et al., 2016). Sciatic nerve transection (SNT) causes muscle atrophy (Higashino et al., 2013), which may result in a decrease in the number of periosteal osteoclasts due to reduced muscle compression (see above) or an increase in the number of periosteal osteoclasts due to unloading/disuse (Suyama et al., 2002). SNT may also result in loss of osteogenic cues released from sensory nerves (Utagawa et al., 2023). To induce muscle and bone atrophy, we performed SNT in the right hindlimb of P16 mice (Fig. 6A), and harvested both SNT and control hindlimbs at P30 (Fig. 6B). Analysis of fibula cross-sections at 3.5 mm from the TFJ confirmed muscle atrophy in the SNT hindlimb (Fig. 6C). In control fibula, the periosteum of the endo-*t*-p cortex, which is anterior side of the fibula, was thinner than that of the peri-*t*-p cortex (Fig. 3E). Moreover, muscle atrophy by SNT treatment increased the thickness of the anterior periosteum, indicating that the SNT-treated fibula has reduced perpendicular compression by the surrounding muscle (Fig. 6D). Osteocytes act as a mechanotransducer in bone matrix (Tatsumi et al., 2007), and E11/podoplanin is upregulated in osteocytes by mechanical loading (Zhang et al., 2006). In the cortex of control fibula, E11 was expressed in osteocytes exhibiting dense dendrites extended towards the bone formation surface [Fig. S4; Cont (L)]. In SNT-treated fibula, E11 expression was lower than in controls, and E11 protein in osteocyte dendrites was barely detectable [Fig. S4; SNT (R)], suggesting that axial mechanical loading is indeed reduced in the SNT model. In the fibula of the control left hindlimb [Cont (L)], osteoclastic TRAP activity was primarily detected at the anterior periosteal surface (arrowheads, Fig. 6E at 3.5 mm, Fig. 6F at 4.5 mm) and at the posterior endocortical surface (arrows, Fig. 6E,F). By contrast, after SNT [SNT (R)], we detected weaker TRAP activity at the periosteum of the anterior cortex corresponding to observed muscle atrophy and, interestingly, TRAP activity spread over the entire circumference of the periosteum (arrowheads, Fig. 6E,F). In untransected control left fibula, osteocalcin-positive osteoblasts were localized to the anterior endocortical surface (paired with periosteal osteoclasts) and to the posterior periosteal surface (paired with endocortical osteoclasts) [Fig. 6G, Cont (L)]. Consistently, after SNT, we detected osteoblasts over the entire circumference of the endocortical surface of the right fibula [Fig. 6G, SNT (R)], suggesting that SNT induces circumferential endo-*t*-p under reduced mechanical loading or nerve injury.

We then determined the direction of bone formation by sequential fluorescence double-labeling with Alizarin Red at P14 (2 days before SNT) and calcein at P28 (2 days before harvest on day 30) (Fig. 6A). At the anterior cortex (Fig. 6H, top) of P30 fibula 4.5 mm above the TFJ, Alizarin staining marking the endosteal surface at P14 (red) was retained in SNT but not control bone at P30, suggesting that periosteal resorption during endo-*t*-p is reduced in SNT bone, possibly owing to reduced muscle compression. This observation is consistent with less active endosteal bone formation seen in SNT versus control bone, as indicated by the smaller distance between calcein at P28 (green) and osteocalcin at P30 (white) in SNT relative to control bone (Fig. 6H, top of panel). Curiously, at the posterior cortex (Fig. 6H, bottom of panel), the positions of Alizarin labeling marking the periosteal surface at P14, calcein labeling at P28 and osteocalcin staining at P30 was reversed in SNT bone (from periosteum to endosteum) relative to control bone (from endosteum to periosteum), indicating that the posterior cortex undergoes a conversion from peri-*t*-p to endo-*t*-p cortex in SNT bone.

These data suggest that SNT decreases endo-*t*-p activity in anterior cortex in a manner suggestive of reduced muscle compression. In addition, SNT induced the circumferential spread of endo-*t*-p since the posterior cortex in SNT exhibited endo-*t*-p, possibly due to reduced axial loading.

### Posterior spreading of cortical canals after sciatic nerve transection

**Fig. 7.**
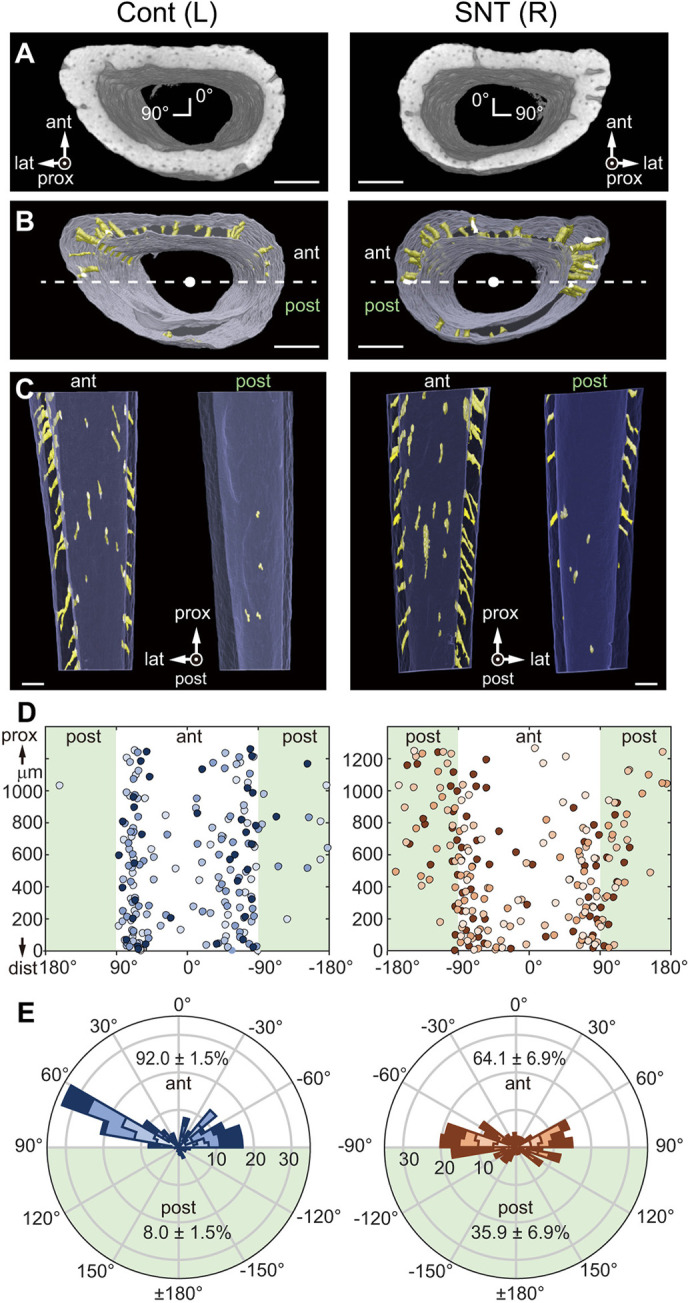
**Circumferential spreading of cortical canals after SNT.** (A) Proximal nano-CT views of control and SNT samples of a 1.27 mm section of fibula centered 4 mm above the tibia-fibula junction (TFJ). Scale bars: 100 µm. (B) Cortical canals (yellow) segmented from nano-CT images shown in A. Canals are spread more widely in SNT versus control fibulae. Scale bars: 100 µm. (C) Anterior (ant) and posterior (post) views of control and SNT samples from bone marrow. The cutting plane at the center of the fibula is the dashed line shown in B. Scale bars: 100 µm. (D) Cortical canal location. Each circle indicates orientation of a cortical canal viewed from the center of the bone marrow cavity on the same proximodistal level as in A plotted against the *z*-axis. Different mice (*n*=4, two female and two male) are distinguished by color saturation. The posterior half of the fibula, which has absolute angles greater than 90°, is highlighted in green. (E) Polar histogram of cortical canal localization shown in D. The percentage of canals located in anterior or posterior halves is also shown. The percentage in the posterior half is significantly higher in SNT(R) than in Cont (L), based on a paired *t*-test (*P*=0.033).

We next determined the location of cortical canals in both left control fibula and right SNT fibula using polar coordinates around the center of the bone marrow cavity and the proximodistal *z*-axis in nano-CT images (Fig. 7A). Cortical canals were visualized by threshold segmentation of cortical bone, which revealed a greater number of posterior canals in SNT relative to control cortical bone (Fig. 7B,C). Bone formation sites visualized by Alizarin injection at P22 revealed that extensive bone formation occurred at cortical canals in the control fibula (Fig. S5). By contrast, in SNT fibula, cortical canals were poorly labeled (Fig. S5). These data suggest that large numbers of transcortical canals are retained in fibulae after SNT, as in-filling of canals is reduced. Plotting of canal positions based on orientation and the *z*-axis revealed more canals in the SNT than in the control fibular cortex in the posterior half (Fig. 7D, highlighted in green). Analysis of polar histograms confirmed posterior spreading of cortical canals from 8% in controls to 35% in the SNT fibular diaphysis (Fig. 7E). These data demonstrate a tight association of endo-*t*-p with cortical canals, even in the context of SNT.

### Cortical canals serve as a cellular pathway supplying preosteoblasts from the periosteum to the endosteum in the endo-*t*-p cortex

**Fig. 8.**
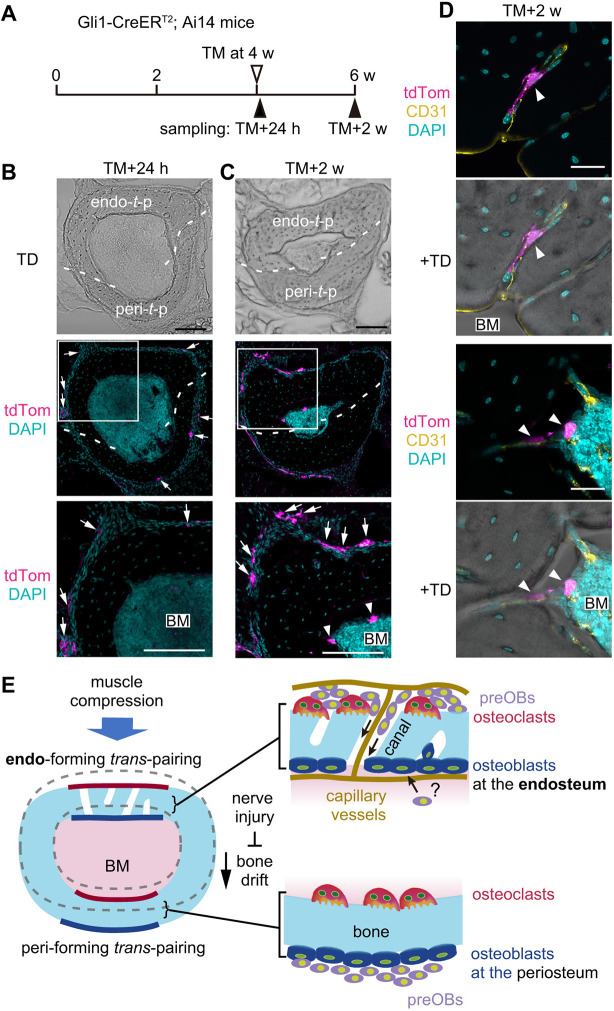
**Cortical canals serve as a cellular pathway supplying preosteoblasts from the periosteum to the endosteum in the endo-*t*-p cortex.** (A) Schematic showing timeline of tamoxifen injection and sampling of Gli1-CreER^T2^; Ai14 mice. Female and male mice were collected at early (TM+24 h) and late (TM+2 w) timepoints. TM, tamoxifen. (B,C) Representative cross-sectional images of fibula at early (B) and late (C) timepoints after tamoxifen administration. Dashed lines indicate the boundary between endo- and peri-*t*-p cortices. Top images (TD for transmitted light differential interference contrast) and the middle images (confocal) were obtained from the same section. The bottom images are magnified views of areas outlined in the middle images showing the endo-*t*-p cortex. tdTomato-positive cells are visible at the periosteum (arrows) and at the endosteum (arrowheads). BM, bone marrow. Scale bars: 100 µm. (D) Localization of tdTomato-positive cells (magenta, arrowheads) and endothelial cells (yellow, CD31-positive) in the endo-*t*-p cortex of fibula at the late timepoint. Scale bars: 20 μm. (E) Summary of *trans*-pairing during bone modeling or bone drift. Bone drift occurs when endo- and peri-*t*-p occur simultaneously. Cortical bone undergoing periosteal bone resorption and endocortical bone formation, i.e. endo-*t*-p, exhibits more and larger cortical canals than bone undergoing peri-*t*-p. Cortical canals, which serve as pathways for capillary vessels, may also facilitate endocortical bone formation by supplying preosteoblasts (preOBs) from the periosteum to the endosteum.

Finally, we examined whether the cortical canals function as a pathway supplying osteoblast progenitor cells from the periosteum to the endosteum. We analyzed the fibular diaphysis of Gli1-CreER^T2^; Ai14 mice by administering tamoxifen at 4 weeks of age in order to perform lineage tracing of osteoblast progenitors (Fig. 8A), as postnatal Gli1-positive osteoprogenitors were localized in the periosteum of the femur and tibia (Shi et al., 2017; Jeffery et al., 2022). In this system, both Gli1-positive osteoprogenitors and their descendants are marked by expression of the red fluorescent protein tdTomato. At an early time point ∼24 h after tamoxifen administration, tdTomato-positive cells were found in the periosteum, but not in the endosteum, of the fibular diaphysis (Fig. 8B, arrows). Two weeks later, tdTomato-positive cells were increased at the periosteum and were also detected at the endosteum of the endo-*t*-p cortex (Fig. 8C, lower panel). Magnified images of cortical canals revealed tdTomato-positive cells and CD31-positive vessels between the periosteum and endosteum (Fig. 8D, arrowheads). These results suggest that osteoblast progenitors derived from the periosteum migrate through cortical canals to promote bone formation at the endosteum in the endo-*t*-p cortex (Fig. 8E).

## DISCUSSION

Bone modeling or drift is regulated by diverse mechanisms, including *trans*-pairing, which occurs between periosteal and endosteal surfaces via coordinated bone resorption and formation activity (Enlow, 1962; Guenther et al., 2008; Maggiano et al., 2015). By contrast, bone can form sequentially in *cis* on the same surface after bone resorption during bone remodeling (Lindsay et al., 2006; Ma et al., 2006; Riggs and Parfitt, 2005). In bone modeling, bone resorption and formation can also occur in *trans* on two opposing surfaces of developing cortical bone. The phenomenon described here as *trans*-pairing has also been reported in humans and other large animals exhibiting osteons or a Haversian system (Montoya-Sanhueza and Chinsamy, 2017; Rauch et al., 2006). However, in terms of mechanism, there have been only a few studies of *trans*-pairing (Edamoto et al., 2019).

Skeletal muscles are major contributors to long bone shape during development. Long bones in mutant mice unable to contract skeletal muscle lose adult bone characteristics, such as curvature, sufficient shaft diameter and traction epiphyses where muscles attach (Gomez et al., 2007; Sharir et al., 2011). The developing mouse fibula is surrounded by muscles such as the FDL or Pt (Charles et al., 2016; Moustafa et al., 2009) that compress the anterior surface of the proximal fibula in a direction away from the tibia as muscles grow between the fibula and tibia. The posterior surface of the distal fibula close to the TFJ is largely compressed towards the tibia by muscles.

Our study suggests that muscle compression induces RANKL (*Tnfsf11*) expression in the periosteum of the cortex undergoing endo-*t*-p during development. Specifically, *in situ* hybridization revealed that cells in the inner layer (cambium) of the periosteum, which faces muscle, express *Tnfsf11*, suggesting that osteoprogenitor cells in the periosteum *per se* may serve as mechanosensors, although osteocytes, periosteal blood capillaries and nerves may also have a mechanosensory function. Our observation of abundant periosteal *Tnfsf11* expression is unexpected because in alveolar bone, osteocytes reportedly produce RANKL in response to mechanical compression (Shoji-Matsunaga et al., 2017). We measured periosteum thickness and found that the periosteum was thinner at endo-*t*-p than peri-*t-*p cortices at all ages examined. In addition, muscle atrophy caused by SNT treatment increased the periosteum thickness at the endo-*t*-p cortex. These data suggest that muscles perpendicularly compress the cortex of the fibula under the physiological development.

Previously, we reported that compression of the cranial base bone by the growing brainstem induces bone resorption on the lagging drift cortex and bone formation on the leading drift cortex (Edamoto et al., 2019). The biological function of compression-induced periosteal bone resorption followed by endocortical bone formation is to provide space for neighboring organs without loss of cortical thickness. The mechanism of *trans*-pairing may confer the ability to undergo flexible modification to bone based on the shape of surrounding soft tissues.

Structural features useful to distinguish between peri- and endo-*t*-p are difficult to detect without 3D structural analysis at high resolution. Our phase-contrast X-ray tomographic microscopy unequivocally revealed that more and larger canals were present in cortical bone undergoing endo- relative to peri-*t*-p, at least in the developing fibulae. We propose that morphological study of cortical bones requires consideration of the concept of *trans*-pairing.

SNT is an experimental procedure used to induce skeletal muscle and bone atrophy/disuse (Higashino et al., 2013; Piet et al., 2019). Indeed in our SNT model, osteocytic E11 expression indicative of mechanical loading was reduced, and muscle atrophy was induced. Importantly, SNT reduced endo-*t*-p activity at the cortex compressed by muscles, as judged by reduced TRAP activity and bone labeling. In addition, SNT induced circumferential bone resorption at the periosteum, and endocortical bone formation also became circumferential, suggesting that periosteal bone resorption induces pairwise endocortical bone formation. Most importantly, cortical canals became circumferential after SNT. Concomitant circumferential spreading of endo-*t*-p and cortical canals suggests a tight association of these events. As cortical porosity increases in aging animals and humans (Piemontese et al., 2017), endo-*t*-p may function in age-related osteoporosis.

How mechanistically do cortical canals contribute to periosteal bone resorption-induced activation of endocortical bone formation? One possibility is that periosteal osteoclasts may activate osteoblast progenitors via diffusible or membrane-bound factors in a manner comparable to ‘coupling factors’ (Henriksen et al., 2014; Kim and Koh, 2019; Matsuo and Irie, 2008) that pass through cortical canals. Alternatively, findings reported here strongly suggest that cortical canals allow preosteoblasts to travel from the periosteum, which is a reservoir of skeletal stem cells, to the endocortical surface. That conclusion is based on three observations. First, we observed *Col1a1*-positive preosteoblasts localized to the periosteum where osteoclasts were found. Consistently, periosteal cells exhibit significant osteogenic activity (Duchamp de Lageneste et al., 2018; Thompson et al., 2016), and they, along with bone marrow cells, reportedly mediate cortical bone formation in growing mouse bone (Debnath et al., 2018; Mizoguchi et al., 2014). Second, we observed preosteoblasts within cortical canals, and periosteum-derived Gli1-positive cells were present in cortical canals and endosteum at the endo-*t*-p cortex. Third, only *Col1a1*-highly positive mature osteoblasts – not *Col1a1*-positive preosteoblasts – were localized on the endosteum of the cortex at a time when endo-*t*-p was occurring. Based on these findings, we propose the model shown in Fig. 8E, whereby preosteoblasts moving through cortical canals promote endocortical bone formation.

Previous studies concluded that periosteal cells make minimal contributions to endocortical bone formation (Debnath et al., 2018; Jeffery et al., 2022), findings that seemingly describe peri-*t*-p cortex. Our analysis distinguishing between endo- and peri-*t*-p cortices, however, suggests that periosteal preosteoblasts can participate in endosteal bone formation by traveling through cortical canals in the endo-*t*-p cortex. Further analyses of communication between peri- and endosteum through cortical canals will provide deeper insights into endo-*t*-p. Compression-induced bone resorption and paired bone formation across the cortex may be a general mechanism underlying bone morphogenesis.

## MATERIALS AND METHODS

### Mice

Mice (*Mus Musculus* C57BL/6J strain) were purchased from CLEA Japan (Tokyo, Japan). Generation of *Col1a1*-AcGFP mice has been previously described (Matsuo et al., 2015). TRAP-tdTomato mice were a kind gift from Dr Masaru Ishii (Osaka University, Japan) (Kikuta et al., 2013). Gli1-CreERT2 [Gli1tm3 (cre/ERT2) Alj/J (JAX007913; Ahn and Joyner, 2004)] and Ai14 [B6.Cg-Gt(ROSA)26Sortm14(CAG-tdTomato)Hze/J (JAX007914; Madisen et al., 2010)] mice were purchased from the Jackson Laboratory. To induce tdTomato expression, Gli1-CreERT2; Ai14 mice were intraperitoneally injected with 150 mg/kg body weight tamoxifen (Sigma-Aldrich) at 4 weeks of age. All animal experiments were approved by the Institutional Animal Care and Use Committee of the Keio University School of Medicine (16-024-26) or the Tokyo Dental College (244103).

### Wholemount TRAP staining

Bones were fixed in 4% paraformaldehyde (PFA)/phosphate-buffered saline (PBS) for 4-10 min and washed in distilled water. TRAP activity was visualized using a TRAP staining kit (387A, Sigma). After washing in distilled water, bones were observed under a Stereo Microscope (Leica, M205 FA). Both female and male mice were used in the experiment.

### Paraffin sections

Lower hindlimbs were skinned and fixed in 4% PFA/PBS overnight at 4°C, and then decalcified in 10% EDTA in 0.1 M Tris (final pH 7) for 14 days at 4°C. Samples were dehydrated, embedded in paraffin and sectioned at 4 µm. Muscles and bones were visualized with Hematoxylin and Eosin staining, and osteoclasts were detected using a TRAP staining kit (387A, Sigma). At least two male mice were used in the same experiment.

### Frozen sections

For calcified bone sectioning, tibia and fibula were fixed in 2% PFA in PBS overnight at 4°C, embedded and sectioned at a thickness of 10 µm using Kawamoto's film method (Kawamoto, 2003; Sakamoto et al., 2017). Calcified frozen sections of the lower leg were permeabilized with 0.2% Tween-20/PBS, and non-specific binding was blocked with 5% normal donkey serum and 1% bovine serum albumin/PBS. For thick sections (50 µm), lower hindlimbs were fixed in 4% PFA in PBS overnight at 4°C, decalcified in 10% EDTA in 0.1 M Tris (final pH 7) for 3 days at 37°C, and delipidated with CUBIC-L (Tainaka et al., 2018) for 1 day at 37°C. Tissues were immersed into 20% sucrose (Fujifilm wako) and 2% polyvinylpyrrolidone K90 (PVP, Fujifilm wako) overnight at 4°C, and frozen in a 8% porcine gelatin (Sigma), 20% sucrose and 2% PVP solution (Kusumbe et al., 2014). Frozen blocks were sectioned by cryostat (LeicaCM3050S). Thick sections were treated with 1 µg/ml proteinase K for 5 min, and non-specific binding was blocked with 5% normal donkey or goat serum, 10 µg/ml donkey or goat IgG and 1% bovine serum albumin/PBS. Sections were then incubated overnight at 4°C with the following primary antibodies: rat anti-osteocalcin monoclonal (R21C-01A, Takara, 1:800), goat anti-MMP9 polyclonal (AF909, R&D, 1:160), Armenian hamster anti-PECAM-1/CD31 (MAB1398Z, Merck, 1:500), goat anti-tdTomato (AB8181-200, Origene, 1:1500) and rabbit anti-RFP polyclonal (PM005, MBL, 1:500). Sections were washed in PBS and incubated with 1μg/ml DAPI (D9542, Sigma-Aldrich) and secondary antibodies: Alexa Fluor 488 donkey anti-rat IgG (A-21208, ThermoFisher, 1:1000), Alexa Fluor 647 donkey anti-goat IgG (A-21447, ThermoFisher, 1:500), Alexa Fluor 647 goat anti-Armenian hamster IgG (ab173004, Abcam, 1:500), Alexa Fluor plus 555 donkey anti-goat IgG (A32732, ThermoFisher, 1:1000) and Alexa Fluor 568 goat anti-rabbit IgG (A-11036, ThermoFisher, 1:500). Images were obtained by confocal microscopy (LSM710, Zeiss; FV4000, Olympus). Both female and male mice were used in the experiment.

### Measurement of periosteal thickness

Paraffin-embedded cross-sections were prepared from mouse fibula at 2-2.5 mm (P9), 2.5-3 mm (P16), 3-3.5 mm (P23) and 3-3.5 mm (P30) above the TFJ. Periosteal thickness was measured using InteredgeDistance macro for ImageJ/Fiji. Note that the interosseous membrane was excluded from this analysis.

### Lightsheet microscopy

DyLight 649 *Lycopersicon esculentum* (tomato) lectin (DL-1178, Vector) was injected (150 µg/mouse) via the orbital plexus to stain the vasculature. Thirty minutes later, lower hindlimbs were removed and fixed overnight in 4% PFA/ PBS at 4°C. Tibiae and fibulae isolated with muscle were delipidated by CUBIC-L at 37°C for 3 days (Tainaka et al., 2018), and decalcified for 3 days in 10% EDTA in 0.1 M Tris (final pH 7) at 37°C. Samples were again incubated in CUBIC-L at 37°C for 3 days and then cleared using a 1:1 water-diluted CUBIC-R+ (T3741, Tokyo Chemical Industry) solution at 37°C for 1 day and then undiluted CUBIC-R+ for 2 days on a shaker. Cleared samples were observed by lightsheet microscopy with a 5× dry lens (Lightsheet Z.1, Zeiss). 100 µm projection images were generated using IMARIS software (Oxford instruments). Both female and male mice were used in the experiment.

### *In situ* hybridization

Isolated samples were fixed with G-Fix (STF-01, GenoStaff) and decalcified with G-Chelate Mild (GCM-01, Genostaff). Paraffin processing was performed with Cell & Tissue Processor CT-Pro20 (GenoStaff) using ethanol and G-NOX. *Tnfsf11* (RANKL) expression was detected in 5 µm paraffin-embedded sections by RNAscope 2.5 HD Reagent kit-BROWN (322300, ACD). Reagents used included a Mm-*Tnfsf11* probe (410921, ACD), dihydrodipicolinate reductase (DapB) (310043, ACD) as a negative control and DNA-directed RNA polymerase II subunit RPB1 (POLR2A) (312471, ACD) as a positive control. *Col1a1* expression was evaluated as described previously (Kuroda et al., 2021), using digoxigenin (DIG)-labeled RNA probes (mouse *Col1a1* sense and antisense; GenoStaff) diluted in G-Hybo-L buffer (Genostaff).

### Sciatic nerve transection

Denervation was performed on the right hindlimb and sham surgery on the left in P16 mice under isoflurane anesthesia. The sciatic nerve was exposed through an incision in the dorsal skin, and an approximately 2 mm long segment was resected at the femoral level. The skin incision was closed using 4-0 nylon sutures. Lower hindlimbs were dissected at P30. Fluorescence double-labeling was performed by subcutaneous injections of 30 mg/kg body weight Alizarin complexone (Sigma-Aldrich) at P14 and of 16 mg/kg body weight calcein (Dojindo) at P28.

### Micro-computed tomography

After removing gastrocnemius and soleus muscles, entire lower hindlimbs were quick-frozen in liquid nitrogen, and then thawed, fixed overnight at 4°C in 4F1G fixative (4% PFA and 1% glutaraldehyde in PBS) and stained with 3.75% Lugol's solution (I_2_KI) in water. Samples were scanned using the R_mCT2 micro-computed tomography (micro-CT) system (Rigaku) at 512 projections/360° at 90 kV and 200 μA. Voxel size for images of whole tibiae and fibulae was 10 μm. Digital segmentation of muscles was performed using TRI/3DBON imaging software (Ratoc System Engineering).

### Phase-contrast X-ray tomographic microscopy

Quantitative phase-contrast X-ray tomographic microscopy with X-ray Talbot interferometry was performed at beamline BL37 of the SPring-8 synchrotron radiation facility (Super Photon ring-8 GeV, Hyogo, Japan) (Takano et al., 2020). A monochromatic X-ray beam (9 keV) was used for tomography, typically at 2.5 s/projection and 720 projections/180°. A differential phase image at every projection was obtained through fringe scanning measurement with five steps (0.5 s exposure per step). X-ray imaging optical magnification was 74; voxel size was 184 nm.

### Nano-computed tomography (nano-CT)

An air-dried fibula was mounted onto an aluminum bar of 3 mm diameter using double-sided tape and then placed on the sample stage of the nano3DX X-ray microscope (Rigaku, Tokyo, Japan) (Kunishima et al., 2022), which is equipped with a quasi-parallel X-ray beam setting and a 2048×2048 pixel (6.5 μm/pixel, 16 bit) sCMOS detector (Zyla 4.2). The fibular mid-diaphysis, centered 4 mm above the TFJ, was scanned by 17 keV X-rays from a Mo-target anode (50 kV, 24 mA). A total of 600 projections were collected per 180° with a pixel size of 1.27 μm (L0540 lens, binning 2) in continuous scan mode, with an exposure time of 4.8 s per frame (60 min in total), and with a sample-to-detector distance of 0.5 mm. The field of view used was 1.33 mm×1.33 mm.

### Software

For image analysis, TRI/3DBON (FCS64, Ratoc System Engineering), Imaris (version 9.7, Oxford Instruments) and ZEN (ZEISS Efficient Navigation, Zeiss) software were used. A cylindrical panoramic image of the fibula was generated by rotating the fibula around the central longitudinal axis at 20° steps in TRI/3DBON. To show central areas, the resulting 18 images of endocortical surfaces were cropped to one quarter of their original size using ImageJ (version 1.53) distributed by Fiji (Schindelin et al., 2012). A cylindrical panoramic image including duplicated anterior regions was then generated by stitching cropped images using Photoshop (Adobe). Cortical canals were segmented by thresholding from cortical bone. ImageJ was used to calculate cross-sectional diameter of cortical canals, which was defined as the minor axis of the best-fitting ellipse. The center of gravity of each canal was determined by Imaris. A line through the centers of gravity of the bone marrow cavity in the first (most proximal) and last (the most distal) slice was used as the *z*-axis. Orientation of the center of gravity of each cortical canal viewed from the center of the bone marrow cavity was calculated at the same *z*-level using MATLAB (R2022a). Scatter plots and polar histograms were plotted using MATLAB.

### Hex nut implantation

C57BL/6J mice (P24) were anesthetized with an intraperitoneal injection of a mix of medetomidine (0.3mg/kg), midazolam (4mg/kg) and butorphanol (5mg/kg). After shaving head fur, the skin was disinfected with 70% ethanol, a small sagittal incision was made to expose the skull and a hex nut (5 mm wide across flats, 2 mm thick, 265 mg) wrapped in parafilm was inserted onto a calvaria through the incision, which was then closed with stitches.

### Clearing calcified tissue with ethyl cinnamate

Bone tissues were fixed in 4% PFA in PBS overnight at 4°C, and then dehydrated using 50% (once), 70% (once) and 100% ethanol (twice) for 1 day each at 4°C. Finally, samples were cleared with ethyl cinnamate (112372, Sigma-Aldrich) at room temperature for 1 day.

## Supplementary Material



10.1242/develop.202194_sup1 Supplementary information
