# Supplementary material for: Developing long bones respond to surrounding tissues by trans-pairing of periosteal osteoclasts and endocortical osteoblasts
Source: Development. 2024 Sep 5;151(17):dev202194. doi: 10.1242/dev.202194 (PMC11423808; doi:10.1242/dev.202194)
Supplement: Supplementary information [file develop-151-202194-s1.pdf]

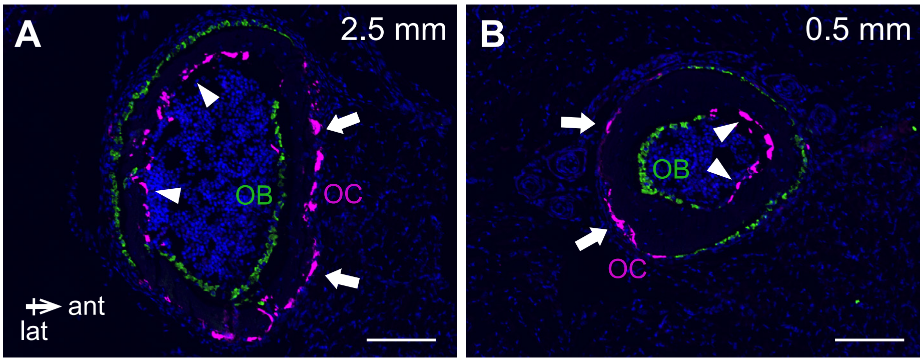


**Fig. S1. Distribution of osteoclasts and osteoblasts in fibula.**
(A, B) Fibula cross-sections at 2.5 mm (A) and 0.5 mm (B) proximal to the TFJ in a P9 TRAP-tdTomato mouse. Magenta, TRAP-tdTomato-positive osteoclasts. Green, osteocalcin-positive osteoblasts. Scale bars, 100 µm. Arrows, TRAP-tdTomato positive periosteal surfaces. Arrowheads, TRAP-tdTomato positive endocortical surfaces.

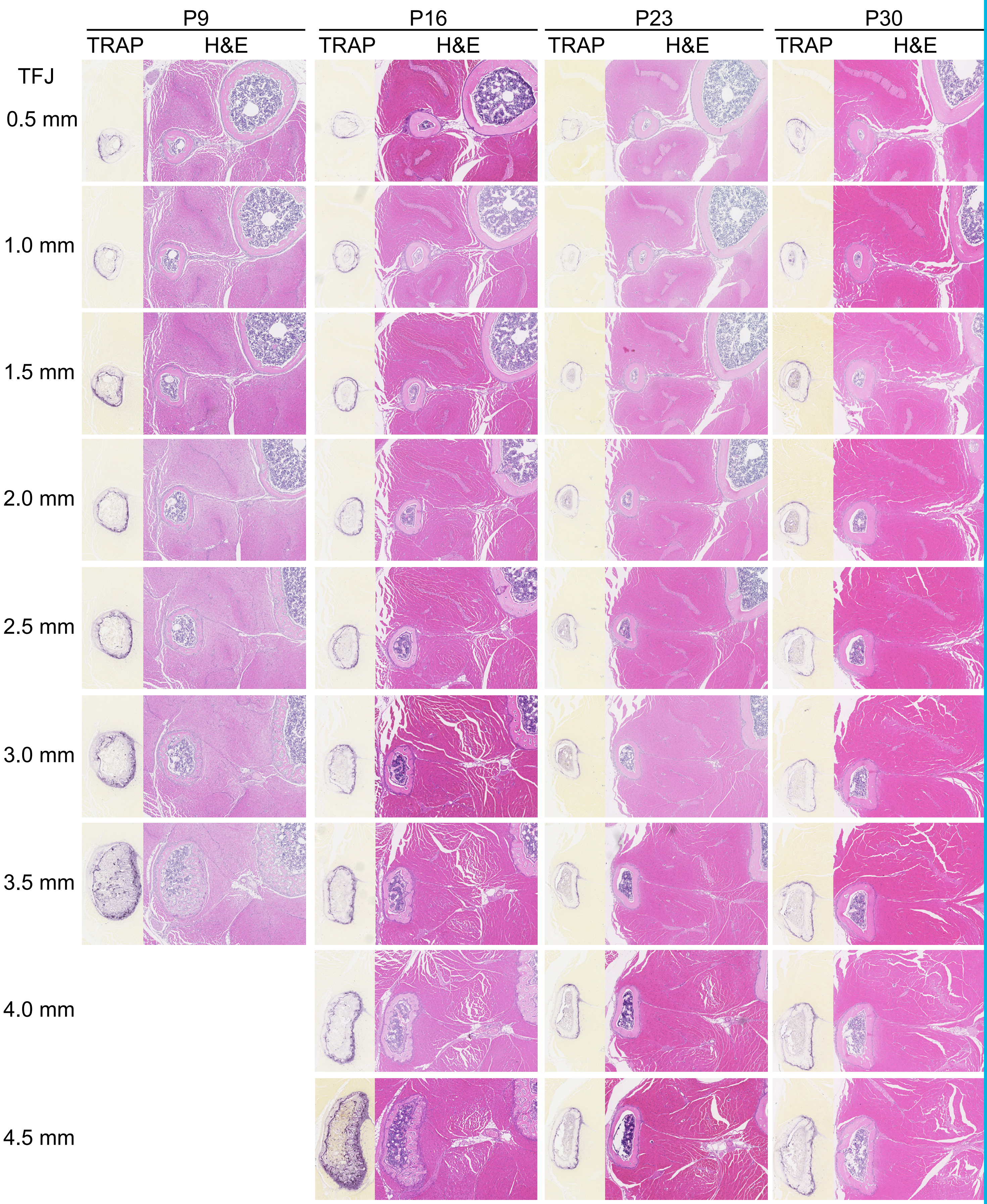


**Fig. S2. Histological changes seen in lower hindlimb during development.** TRAP activity and H&E staining of paraffin cross-sections at 0.5 mm to 4.5 mm from the TFJ in mice at P9, P16, P23, and P30. Scale bars, 1 mm.

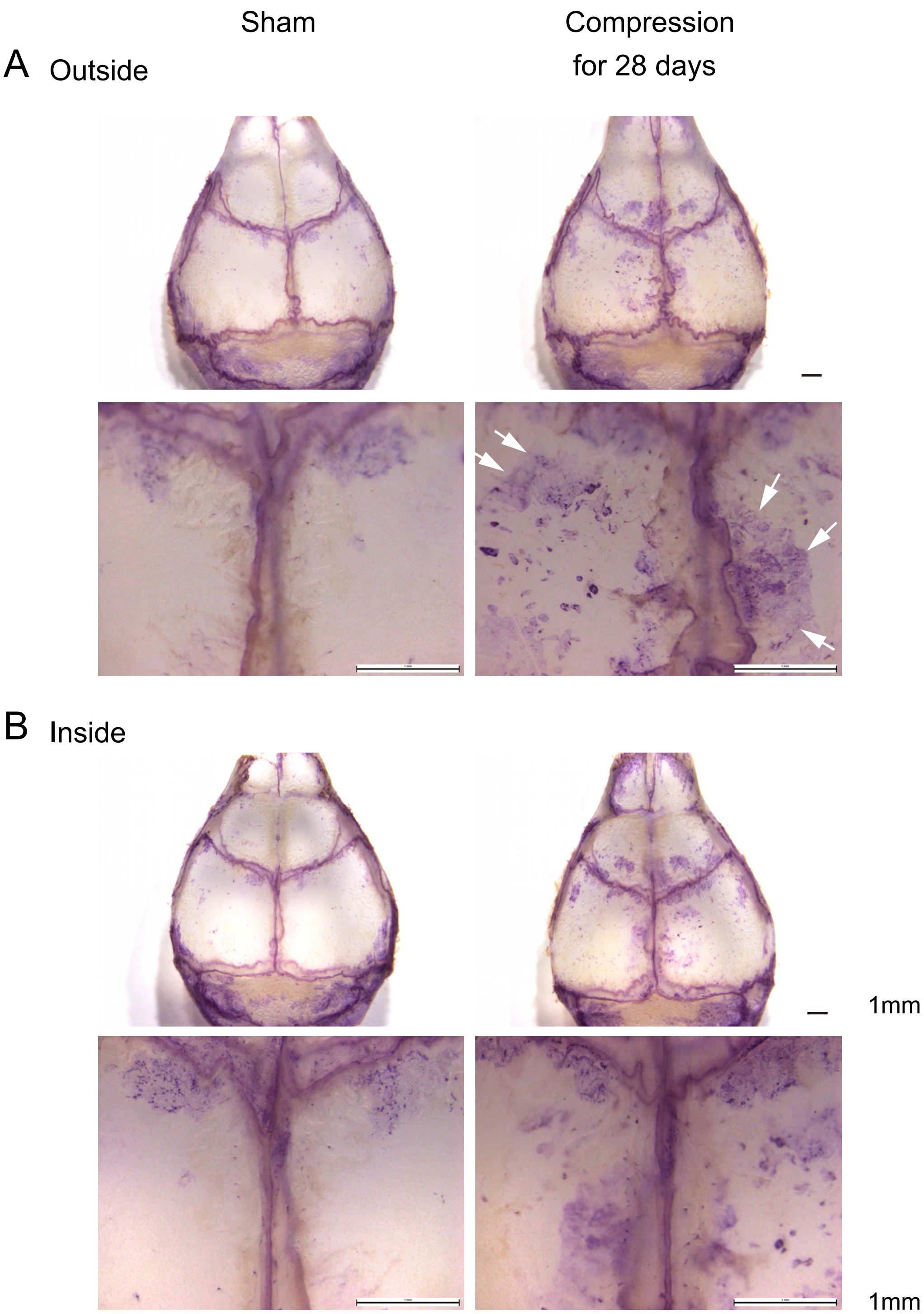


**Fig. S3. Mechanical compression-induced TRAP-activity on calvaria.** Outside (A) and inside (B) views of calvaria after wholemount staining for TRAP activity (purple). Top rows, calvarial surfaces. Bottom rows, higher magnifications. Left, sham operated. Right, compressed for 28 days (from P24-P52). Arrows indicate ectopically induced TRAP-positive resorption surfaces. Note that those areas are located outside (skin side, in focus), rather than inside (brain side, out of focus). Scale bars, 1 mm.

Development • Supplementary information

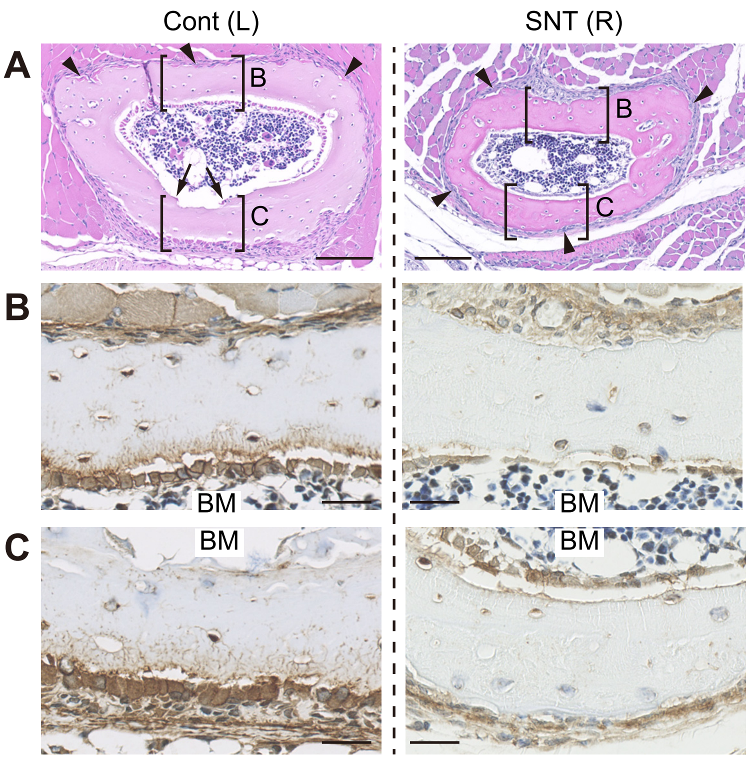


**Fig. S4. E11/podoplanin immunostaining of SNT and control fibulae.** (A-C) Histological analyses of paraffin cross-sections at 3.5 mm from the TFJ of SNT and control hindlimbs described above. (A) H&E staining of fibula. Arrowheads and arrows indicate periosteal and endocortical bone resorption surfaces, respectively. Bracketed regions are analyzed in (B) and (C). Scale bars, 100 µm. (B, C) E11/podoplanin immunostaining of endo-t-p (B) and peri-t-p (C) cortices. Scale bars, 25 µm.

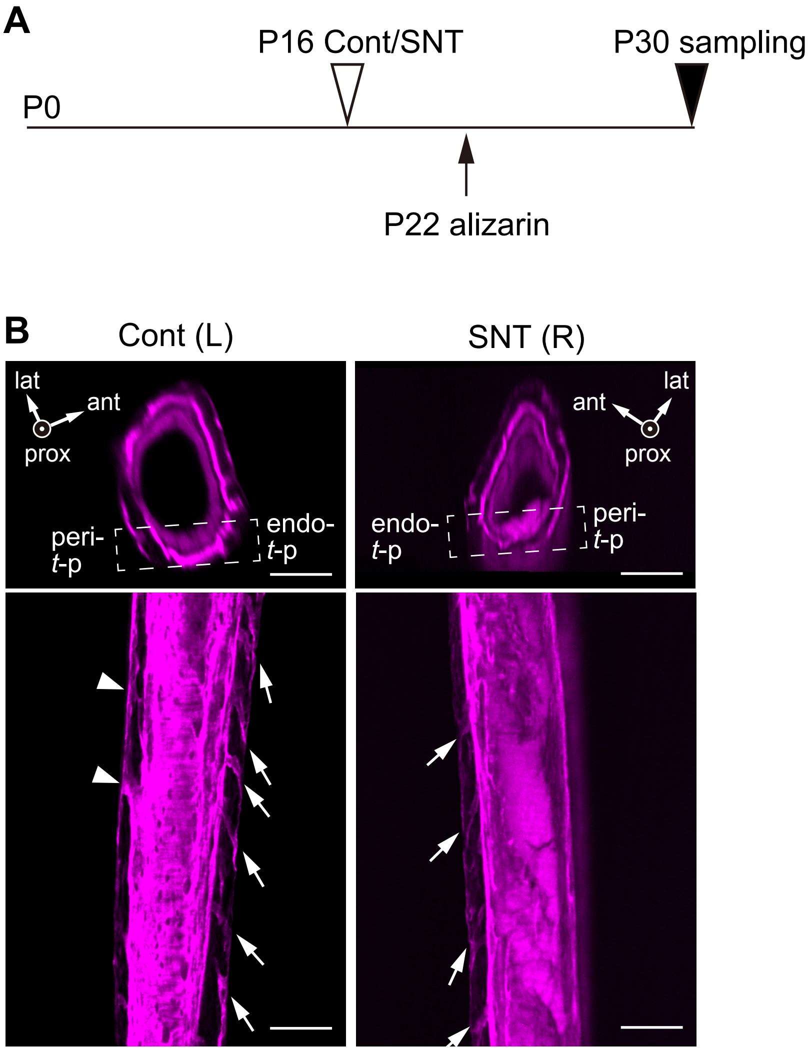


**Fig. S5. SNT decreases intracortical bone formation.**(A) Schematic showing timeline of SNT and bone labeling.(B) Lightsheet fluorescence microscopy detection of alizarin (magenta) labeling in the fibula (at ~3 mm above the TFJ) of control (L) and SNT (R) samples. Areas indicated by dashed lines in upper panels are shown as 100 μm-thick projection images in lower panels. Arrows, intracortical bone labeling. Scale bar, 150 μm.

Development • Supplementary information
