# Supplementary material for: Developing long bones respond to surrounding tissues by trans-pairing of periosteal osteoclasts and endocortical osteoblasts
Source: Development. 2024 Sep 5;151(17):dev202194. doi: 10.1242/dev.202194 (PMC11423808; doi:10.1242/dev.202194)
Supplement: Supplementary file 1 [file develop-151-202194_review_history.pdf]

Developing long bones respond to surrounding tissues by *trans*-pairing of periosteal osteoclasts and endocortical osteoblasts

Yukiko Kuroda, Masaki Yoda, Katsuhiro Kawaai, Motoharu Tatenuma, Toshihide Mizoguchi, Shinichirou Ito, Masataka Kasahara, Yanlin Wu, Hidekazu Takano, Atsushi Momose and Koichi Matsuo
DOI: 10.1242/dev.202194

Editor: Haruhiko Koseki

Review timeline

Original submission:	5 April 2022
Editorial decision:	11 April 2022
Resubmission:	18 July 2023
Editorial decision:	18 August 2023
First revision received:	20 January 2024
Editorial decision:	28 February 2024
Second revision received:	31 July 2024
Accepted:	1 August 2024

Original submission

First decision letter

MS ID#: DEVELOP/2023/200830

MS TITLE: Developing long bones respond to surrounding tissues by *trans*-pairing of periosteal osteoclasts and endocortical osteoblasts

AUTHORS: Yukiko Kuroda, Masaki Yoda, Katsuhiro Kawaai, Motoharu Tatenuma, Yanlin Wu, Hidekazu Takano, Atsushi Momose, and Koichi Matsuo

Dear Dr. Matsuo

Many thanks for submitting your manuscript to Development.

As a requisite step of the editorial procedure, I have read the manuscript to determine if the contents of the study meet the aim and scope of the journal. After reviewing the paper, I recognize that this is a study of the phenomenon of bone remodelling by trans-pairing process of periosteal resorption and endosteal growth in the fibula, a model of adaptive response. The findings showed that trans-paired inward growth of the fibula is accompanied by the concerted osteoclastic rand osteoblast activity in the transcortical plane and that atrophy of the muscle associated with bone, while not affecting the trans-pairing activity, led to disorientation of the activity in the cortical area, and the site of active trans-pairing activity is topographically related to regions of enhanced vascularization. While these are interesting findings that point to the requirement of mechanical constraint (e.g., from compression-exerting muscle) and vascular function (e.g., for bioavailability of morphogen/s), the entry point of the localised biomechanical cue to the mechano-sensing pathway and the downstream molecular network of osteoclastic/osteoblastic activity, and the role of vascularization in mediating the trans-pairing activity remains obscure. Delineation of the mechanistic attribute of mechanical cue and vascular function to the activation of trans-pairing activity, which may require further experimental works, would enhance the merit of this study. The main criterion for publication in Development is that a Research Article should make a significant

© 2024. Published by The Company of Biologists under the terms of the Creative Commons Attribution License (https://creativecommons.org/licenses/by/4.0/).

and novel contribution to our understanding of developmental mechanisms. Studies lacking such a contribution, no matter how meticulous, are not of priority for publication. On these considerations, I am returning the paper without further review.

Resubmission

First decision letter

MS ID#: DEVELOP/2023/202194

MS TITLE: Developing long bones respond to surrounding tissues by *trans*-pairing of periosteal osteoclasts and endocortical osteoblasts

AUTHORS: Yukiko Kuroda, Masaki Yoda, Katsuhiro Kawaai, Motoharu Tatenuma, Yanlin Wu, Hidekazu Takano, Atsushi Momose, and Koichi Matsuo

I have now received all the referees' reports on the above manuscript, and have reached a decision. The referees' comments are appended below, or you can access them online: please go to BenchPress and click on the 'Manuscripts with Decisions' queue in the Author Area.

As you will see, the referees express considerable interest in your work, but have some significant criticisms and recommend a substantial revision of your manuscript before we can consider publication. Particularly, it is essentially important to perform lineage tracking experiments to substantiate your model. If you are able to revise the manuscript along the lines suggested, which may involve further experiments, I will be happy receive a revised version of the manuscript. Your revised paper will be re-reviewed by one or more of the original referees, and acceptance of your manuscript will depend on your addressing satisfactorily the reviewers' major concerns. Please also note that Development will normally permit only one round of major revision. If it would be helpful, you are welcome to contact us to discuss your revision in greater detail. Please send us a point-by-point response indicating your plans for addressing the referees' comments, and we will look over this and provide further guidance.

Please attend to all of the reviewers' comments and ensure that you clearly highlight all changes made in the revised manuscript. Please avoid using 'Tracked changes' in Word files as these are lost in PDF conversion. I should be grateful if you would also provide a point-by-point response detailing how you have dealt with the points raised by the reviewers in the 'Response to Reviewers' box. If you do not agree with any of their criticisms or suggestions please explain clearly why this is so.

Reviewer 1

Advance summary and potential significance to field

The authors study the postnatal development of fibula and tibia and note an increase in osteoclastic resorption in the periosteum that occurs concurrently with an increase in channels connecting the periosteum and endosteum. They suggest that the new bone that forms on the endosteum is generated by preosteoblastsosteoblasts that originated in the periosteum. if this is true, it's an important advance.

Comments for the author

The authors suggest that the new bone formation that they note on the endocortical surface comes from osteoblasts that originated from precursors at the pericondrial surface. The authors note that there are osteoblasts in canals between those surfaces that could make this possible. But they do not show that the endosteal osteoblasts originated from the periosteal precursors (as opposed to marrow precursors). Greenblatt's group published a nice paper (referenced by the authors) in which they identify periosteal precursors of osteoblasts and show that they have a gene signature that distinguishes those cells from marrow osteoblast precursors. (Shawon Debnath, Alisha R. Yallowitz,

© 2024. Published by The Company of Biologists under the terms of the Creative Commons Attribution License (https://creativecommons.org/licenses/by/4.0/).

Jason McCormick, Sarfaraz Lalani, Tuo Zhang, Ren Xu, Na Li, Yifang Liu, Yeon Suk Yang, Mark Eiseman, Jae-Hyuck Shim, Meera Hameed, John H. Healey, Mathias P. Bostrom, Dan Avi Landau, and Matthew B. Greenblatt. Discovery of a periosteal stem cell mediating intramembranous bone formation. Nature. 2018 Oct; 562(7725): 133–139.) Those authors used a cre driver specific for periosteal stem cells to knock out the osterix gene and showed that this disrupted periosteal bone formation but actually increased endocortical bone formation. Certainly, in that case, the endocortical bone formation did not originate from periosteal precursors. There is still much to do to establish the fates of periosteal stem cells, but the current manuscript doesn't mention the Greenblatt lab's experiment that I've just quoted or provide any alternative direct evidence that the endosteal osteoblasts originated in periosteal precursors. So the authors' hypothesis is intriguing and might even be right, but they need much more direct lineage data to show it.

Their demonstration of the canals between the periosteum and endosteum are the highlight of the paper and may well provide the path that the authors postulate.

Reviewer 2

Advance summary and potential significance to field

Kuroda et al present a substantial study into cell behaviors involved in bone growth. Bone is known to adapt its shape through modeling and re-modeling. Modeling is defined as "the formation of bone by osteoblasts or resorption of bone by osteoclasts on a given surface" as opposed to remodeling "in which osteoblast and osteoclast activity occur sequentially in a coupled manner on a given bone surface" ('textbook' definitions from Allen and Burr 2014, doi.org/10.1016/B978-0-12-416015-6.00004-6). Much is known about the molecular mechanisms which locally couple the activity of osteoblasts and osteoclasts. Modeling has for several decades been hypothesized to be orchestrated by osteocytes within the bone matrix (e.g. Lanyon 1993, 10.1007/BF01673415 and many subsequent papers), most importantly during functional adaptation to local mechanical strain. Mice lacking osteocytes or osteocyte-secreted proteins do not lose bone in response to mechanical disuse (e.g. Tatsumi et al Cell Metab 2007). This history is important to interpret the authors' claims including the proposal of "a fundamental mechanism underlying endo-forming transpairing, namely, triggering by periosteal osteoclasts, which then induce endocortical bone formation by enabling periosteal preosteoblasts to pass through cortical canals towards the endosteum."

Comments for the author

1. What is the evidence that endosteal formation is "triggered by periosteal osteoclasts"? None of the data presented demonstrate that periosteal osteoclasts produce a signal which is sensed by osteoblasts and increases their activity endosteally, nor is the role of osteocytes meaningfully examined.
2. "…enabling periosteal preosteoblasts to pass through cortical canals towards the endosteum" – what evidence is provided that pre-osteoblasts migrate through cortical channels in a periosteal to endosteal direction? The Col1a1 localisation data does not provide evidence of directional migration, particularly when closely associated with blood vessels.
3. "Sites of periosteal bone resorption correspond to muscle compression". This statement could just as easily read that "Sites of muscle expansion correspond to space available thanks to bone resorption". The authors could test this proposition, at least correlatively, by determining whether muscle volumetric expansion which might compress bone precedes the onset of resorption. One might also expect greater compaction of cells on the periosteum at sites of proposed compression, which could be quantified in histological sections.
4. The image showing in situ of Tnfsr11 is not of sufficient quality, at least in the PDF available, to exclude its expression by osteocytes. Osteocytes within the image appear to have brown dots (positive signal) in them as well.
5. "preosteoblasts weakly positive for Col1a1 were present above osteoblasts at the periosteaum." Are these different from the previously-described canopy cells, which are also associated with blood vessels (e.g. Andersen et al Am J Pathol 2009)?
6. The sciatic neurectomy experiment is difficult to interpret because it combines the anti-growth effect of disuse on a period of rapid growth. The reviewer's understanding is that formation

© 2024. Published by The Company of Biologists under the terms of the Creative Commons Attribution License (https://creativecommons.org/licenses/by/4.0/).

is indicated by the sequence of red (alizarin) to green (calcein) to white (osteoblast surface 2 days after calcein).
It is not clear why the bottom (lateral) aspect of the neurectomised bone retained alizarin on the periosteal surface – the TRAP staining in 6D shows this is not a quiescent surface. Was it the former endosteal surface which has modelled by endosteal formation as the formely periosteal surface was resorbed (red◊green◊white)? The equivalent alizarin line in the non-neurectomised limb does in fact appear to span the periosteal to endosteal surface. A much more detailed analysis of this data, likely including quantification of inter-label distance for bone formation rates and multi-site comparisons to help interpret drift direction is needed.
7. Sciatic neurectomy decreases muscle volume, yet osteoclast surface per bone surface (inferred from TRAP staining) is not decreased. This argues against the assertion that “osteoclastic bone resorption surfaces are established in response to muscle compression”.
8. Regarding the larger trans-cortical canals following neurectomy: can the authors determine whether disuse stopped in-filling of canals which were large at the start of the experiment or caused expansion of canals which were smaller at the start (e.g. by comparison with canals in the tibiae of mice at P16)?
9. In Figure 4C, the number of mice analysed is not clear. The individual mouse, not multiple canals within the same mouse, should be used as the unit of measure.
10. Were male or female mice used for the experiments?

Minor:
11. The concept of trans-pairing, while interesting, needs to be put in context of what is known about modeling and remodeling in the introduction and discussion particularly in species without Haversian canals.

Reviewer 3

Advance summary and potential significance to field

In this study, the authors have examined the coordinated activity of periosteal and endosteal osteoblasts and osteoclasts in developing long bones. Through a series of in vivo experiments, the authors posit that osteoclast activation at the periosteal surface, potentially due to muscle and/or nerve-induced signals results in the migration of preosteoblasts through cortical canals to the endocortical surface to produce new bone tissue. The experiments are well described, the manuscript itself is well-written, and the figures are clear and compelling. However, more effort needs to be dedicated to ensuring that the conclusions are supported by the data before it is suitable for publication.

Comments for the author

Major Comments:

1. The authors propose that sites of periosteal bone resorption correspond to muscle compression (related to Figures 2 and 3). However, the authors do not perform any experimentation to confirm this hypothesis – the conclusion is based on anatomical location. As a result, the authors should carefully consider the language regarding this conclusion. Specifically, can the authors confirm that these muscles provide compression to the periosteal surfaces in question (vs. proximity with negligible mechanical force, tension, or a more complex stress state)?

2. Using phase-contrast X-ray techniques (Fig 4), the authors nicely illustrate that cortical canals are present primarily on endo-t-p as compared to peri-t-p surfaces. However, it is not clear if the images shown are representative of the data. For example, the Fig. 4B clearly shows about 10 endo-t-p cortical canals (asterisks) and 0 peri-t-p canals. The quantification in Fig. 4C is more even between the groups, with 25 endo-t-p and 14 peri-t-p.
Furthermore, it appears that only two mice were used for this experiment. It would be preferred to either perform more biological replicates or include truly representative images from both mice in the text.

© 2024. Published by The Company of Biologists under the terms of the Creative Commons Attribution License (https://creativecommons.org/licenses/by/4.0/).

3. Sciatic nerve transection (SNT) is used to investigate trans-pairing and drift. However, the primary effect here seems to be related to muscle function / load on the bone rather than skeletal innervation. The authors should comment on this in the discussion as well as modify Figure 8 to include the contribution of nerves and/or muscle to their model.

a. Related to this, the authors reference “inflammatory responses to sciatic nerve transection” on page 10. Since no inflammatory markers were utilized in this study, this phrase should be removed or revised.

4. The authors have provided a series of observations that are consistent with their proposed mechanism (Fig. 8). One perturbation (SNT) is investigated although the mechanism does not directly test any specific aspect of the model.
It may be helpful to perform a positive intervention (e.g. direct application of force to developing bone in a non-physiological direction) to directly show how these mechanisms are controlled.
5. The authors have extensively studied the developing mouse hindlimb specifically the fibula. Were any other bones examined, perhaps superficially (e.g. as in Figure 1), that would illustrate the generalizability of trans-pairing in long bones?

Minor Comments:

1. If possible, information regarding the specific target or stain in each image should appear in the figure itself (e.g. in Fig 5A, the “OB OC” is helpful).

Response to Reviewers

Reviewer 1 Advance Summary and Potential Significance to Field:
The authors study the postnatal development of fibula and tibia and note an increase in osteoclastic resorption in the periosteum that occurs concurrently with an increase in channels connecting the periosteum and endosteum. They suggest that the new bone that forms on the endosteum is generated by preosteoblasts/osteoblasts that originated in the periosteum. if this is true, it's an important advance.

Reviewer 1 Comments for the Author:
The authors suggest that the new bone formation that they note on the endocortical surface comes from osteoblasts that originated from precursors at the pericondrial surface. The authors note that there are osteoblasts in canals between those surfaces that could make this possible. But they do not show that the endosteal osteoblasts originated from the periosteal precursors (as opposed to marrow precursors). Greenblatt's group published a nice paper (referenced by the authors) in which they identify periosteal precursors of osteoblasts and show that they have a gene signature that distinguishes those cells from marrow osteoblast precursors. (Shawon Debnath, Alisha R. Yallowitz, Jason McCormick, Sarfaraz Lalani, Tuo Zhang, Ren Xu, Na Li, Yifang Liu, Yeon Suk Yang, Mark Eiseman, Jae- Hyuck Shim, Meera Hameed, John H. Healey, Mathias P. Bostrom, Dan Avi Landau, and Matthew B. Greenblatt. Discovery of a periosteal stem cell mediating intramembranous bone formation. Nature. 2018 Oct; 562(7725): 133–139.) Those authors used a cre driver specific for periosteal stem cells to knock out the osterix gene and showed that this disrupted periosteal bone formation but actually increased endocortical bone formation. Certainly, in that case, the endocortical bone formation did not originate from periosteal precursors. There is still much to do to establish the fates of periosteal stem cells, but the current manuscript doesn’t mention the Greenblatt lab’s experiment that I’ve just quoted or provide any alternative direct evidence that the endosteal osteoblasts originated in periosteal precursors. So the authors’ hypothesis is intriguing and might even be right, but they need much more direct lineage data to show it.

Their demonstration of the canals between the periosteum and endosteum are the highlight of the paper and may well provide the path that the authors postulate.

© 2024. Published by The Company of Biologists under the terms of the Creative Commons Attribution License (https://creativecommons.org/licenses/by/4.0/).

We agree that the results of Greenblatt's group may contradict our hypothesis and that more direct experimental evidence is needed to confirm that osteoblasts at the endo-forming *trans*-pairing (endo-*t*-p) cortex originate from periosteal precursors.

Greenblatt's and other groups claim that periosteal cells contribute little to endocortical bone formation (Debnath et al., 2018; Jeffery et al., 2022). We feel that future studies focusing on the endo-*t*-p cortex separately from the peri-*t*-p cortex could clarify these matters.

To provide such direct evidence, we used Gli1-CreER^{T2} mice crossed with Ai14 reporter mice (Shi et al., 2017). Use of Gli1-CreER^{T2} mice allows labeling of periosteal skeletal stem cells in the diaphysis of long bones more restrictively than use of Ctsk-Cre mice (Jeffery et al., 2022). Thus, we injected 4-week-old Gli1- CreER^{T2} mice with tamoxifen to label those periosteal precursors, and 2 weeks later detected tdTomato-positive cells at the periosteum of the peri-*t*-p cortex, as expected. By contrast, tdTomato-positive cells of the endo-*t*-p cortex were localized in cortical canals and at the endosteum and periosteum, supporting the hypothesis that periosteal precursors migrate through cortical canals to induce endocortical bone formation. We present this new lineage-tracing data in Fig. S4 of the revision. However, we do not exclude the contribution of marrow-derived cells, and therefore have added marrow-derived preosteoblasts to the model shown in Fig. 8. We also now discuss discrepancies between the Debnath paper (2018) and this study in the revised manuscript.

Reviewer 2 Advance Summary and Potential Significance to Field:
Kuroda et al present a substantial study into cell behaviors involved in bone growth. Bone is known to adapt its shape through modeling and re-modeling.
Modeling is defined as “the formation of bone by osteoblasts or resorption of bone by osteoclasts on a given surface” as opposed to remodeling “in which osteoblast and osteoclast activity occur sequentially in a coupled manner on a given bone surface” (‘textbook’ definitions from Allen and Burr 2014, doi.org/10.1016/B978-0- 12-416015-6.00004-6). Much is known about the molecular mechanisms which locally couple the activity of osteoblasts and osteoclasts. Modeling has for several decades been hypothesized to be orchestrated by osteocytes within the bone matrix (e.g. Lanyon 1993, 10.1007/BF01673415 and many subsequent papers), most importantly during functional adaptation to local mechanical strain. Mice lacking osteocytes or osteocyte-secreted proteins do not lose bone in response to mechanical disuse (e.g. Tatsumi et al Cell Metab 2007). This history is important to interpret the authors’ claims including the proposal of “a fundamental mechanism underlying endo-forming transpairing, namely, triggering by periosteal osteoclasts, which then induce endocortical bone formation by enabling periosteal preosteoblasts to pass through cortical canals towards the endosteum.”

Reviewer 2 Comments for the Author:
1. What is the evidence that endosteal formation is “triggered by periosteal osteoclasts”? None of the data presented demonstrate that periosteal osteoclasts produce a signal which is sensed by osteoblasts and increases their activity endosteally, nor is the role of osteocytes meaningfully examined.

Accordingly, we now state in the Introduction that trans-pairing driven-bone drift occurs in an osteoclast-dependent manner based on previous analyses of osteopetrotic *Tnfsf11-/-* mice and mice injected with anti-RANKL antibody or osteoprotegerin (Edamoto et al., 2019; Piemontese et al., 2017). These mice exhibit impaired endocortical bone formation. Therefore, we propose that periosteal osteoclasts trigger endosteal bone formation.

In the Results we now cite studies reporting that osteocytes act as a mechanotransducer in bone matrix (Tatsumi et al., 2007) and that E11/podoplanin is upregulated in osteocytes by mechanical loading (Zhang et al., 2006). We also performed additional experiments to detect E11 expression in fibular cortex and show that data in Fig. S5AB. That analysis confirmed that gene expression in osteocytes is altered in response to axial mechanical loading in the SNT model, as expected. However, E11 expression did not response to muscle compression, because there were no differences in osteocytic E11 expression between endo- and peri-*t*-p cortices in developing long bone.
2. “…enabling periosteal preosteoblasts to pass through cortical canals towards the endosteum” –

© 2024. Published by The Company of Biologists under the terms of the Creative Commons Attribution License (https://creativecommons.org/licenses/by/4.0/).

what evidence is provided that pre-osteoblasts migrate through cortical channels in a periosteal to endosteal direction? The Col1a1 localisation data does not provide evidence of directional migration, particularly when closely associated with blood vessels.

Based on this comment, we performed lineage-tracing experiments using Gli1- CreER^{T2} mice crossed with Ai14 reporter mice. Chasing tdTomato-positive cells after tamoxifen administration to 4-week-old mice allowed us to lineage-trace periosteal Gli1-positive osteoblast progenitors at cortical bone (Shi Y et al., 2017). Two weeks after tamoxifen injection, we detected tdTomato-positive cells at the peri-*t*-p cortex in the periosteum. By contrast, tdTomato-positive cells of the endo-*t*- p cortex localized in cortical canals and the endosteum and periosteum. These data support our hypothesis that periosteal osteoblasts travel through cortical canals towards the endosteum in the endo-*t*-p cortex. Thus, although the contribution of periosteal progenitors to cortical bone formation in the endo-*t*-p was indicated, the directional migration of *Col1a1* osteoblasts remains unknown.
Therefore, we have modified our model in Fig.8 to avoid excluding the possibility that osteoblast progenitors are also supplied from the bone marrow. These results are presented in Fig. S4 and Fig. 8 in the revision.

3. “Sites of periosteal bone resorption correspond to muscle compression”. This statement could just as easily read that “Sites of muscle expansion correspond to space available thanks to bone resorption”. The authors could test this proposition, at least correlatively, by determining whether muscle volumetric expansion which might compress bone precedes the onset of resorption. One might also expect greater compaction of cells on the periosteum at sites of proposed compression, which could be quantified in histological sections.

Accordingly, we measured periosteum thickness at both endo- and peri-*t*-p cortices of mouse fibula at P9, P16, P23 and P30 (Fig. S2), and found that the periosteum was thinner at the endo-*t*-p than the peri-*t*-p cortex in all ages examined. These results suggest that the periosteum of endo-*t*-p is more compressed than that of peri-*t*-p and that muscle expansion induces periosteal compaction. These new data are shown in revised Figure 3E and described in the Results and Discussion.

4. The image showing in situ of *Tnfsr11* is not of sufficient quality, at least in the PDF available, to exclude its expression by osteocytes. Osteocytes within the image appear to have brown dots (positive signal) in them as well.
In response to this comment, we replaced *Tnfsf11* in situ images with higher magnification images. As shown in revised Figure 3F and G, brown dots (positive signals) were most frequently observed in the cambium layer but were also detected in some osteocytes and in bone marrow.

5. “preosteoblasts weakly positive for Col1a1 were present above osteoblasts at the periosteum.” Are these different from the previously-described canopy cells, which are also associated with blood vessels (e.g. Andersen et al Am J Pathol 2009)?

“Canopy cells” were previously reported at the endosteal surface in human bone (Andersen et al, J. Pathol, 2009). Although we observed canopy cells covering osteoclasts and osteoblasts in mouse long bone, they were *Col1a1*-negative. We conclude that periosteal cells weakly positive for *Col1a1* differ from canopy cells.

6. The sciatic neurectomy experiment is difficult to interpret because it combines the anti-growth effect of disuse on a period of rapid growth. The reviewer’s understanding is that formation is indicated by the sequence of red (alizarin) to green (calcein) to white (osteoblast surface 2 days after calcein). It is not clear why the bottom (lateral) aspect of the neurectomised bone retained alizarin on the periosteal surface – the TRAP staining in 6D shows this is not a quiescent surface. Was it the former endosteal surface which has modelled by endosteal formation as the formely periosteal surface was resorbed (red◊green◊white)? The equivalent alizarin line in the non-neurectomised limb does in fact appear to span the periosteal to endosteal surface. A much more detailed analysis of this data, likely including quantification of inter-label distance for bone formation rates and multi- site comparisons to help interpret drift direction, is needed.

© 2024. Published by The Company of Biologists under the terms of the Creative Commons Attribution License (https://creativecommons.org/licenses/by/4.0/).

We agree with the reviewer's interpretation of the histological analysis and now describe double-labeling and osteocalcin immunostaining in greater detail.
Moreover, we now analyze paraffin sections (using both H&E and TRAP staining) in addition to frozen sections to identify drift directions.

7. Sciatic neurectomy decreases muscle volume, yet osteoclast surface per bone surface (inferred from TRAP staining) is not decreased. This argues against the assertion that "osteoclastic bone resorption surfaces are established in response to muscle compression".

Inspired by the reviewer, we now detect weaker TRAP activity at the periosteum of the anterior cortex after SNT corresponding to observed muscle atrophy. We described this in Results and Discussion in revision.

8. Regarding the larger trans-cortical canals following neurectomy: can the authors determine whether disuse stopped in-filling of canals which were large at the start of the experiment or caused expansion of canals which were smaller at the start (e.g. by comparison with canals in the tibiae of mice at P16)?

We agree that it would be interesting to determine whether the larger cortical canals were initially large, or whether small canals expand to become large. Analysis of a disuse muscle atrophy model with tail-suspension experiments is one approach, and indeed we have submitted an animal study protocol to the Institutional Animal Care and Use Committee (IACIC) to conduct such analyses in the future. However, here, to address the reviewer's question, we performed additional bone labeling experiments on fibulae with or without SNT and conducted lightsheet microscopy analysis (Fig. S6). In SNT-treated fibula, intracortical labeling was decreased compared with control fibula. These data suggest that the increase in cortical canal number was due to stopping the in-filling of canals.

9. In Figure 4C, the number of mice analysed is not clear. The individual mouse, not multiple canals within the same mouse, should be used as the unit of measure.

Accordingly, we increased the number of mice from 2 to 4 and now indicate this in the Figure 4C legend. We also now show the number of canals per bone surface area (canal density) of individual mice in Figure 4D in the revision.

10. Were male or female mice used for the experiments?

We now provide the number of male and female mice analyzed (underlined) in each figure legend.

Minor:
11. The concept of trans-pairing, while interesting, needs to be put in context of what is known about modeling and remodeling in the introduction and discussion, particularly in species without Haversian canals.

Thank you for this comment. Accordingly, we added an explanation of modeling and remodeling to the revised Introduction and Discussion.
Reviewer 3 Advance Summary and Potential Significance to Field:
In this study, the authors have examined the coordinated activity of periosteal and endosteal osteoblasts and osteoclasts in developing long bones. Through a series of in vivo experiments, the authors posit that osteoclast activation at the periosteal surface, potentially due to muscle and/or nerve-induced signals, results in the migration of preosteoblasts through cortical canals to the endocortical surface to produce new bone tissue. The experiments are well described, the manuscript itself is well-written, and the figures are clear and compelling. However, more effort needs to be dedicated to ensuring that the conclusions are supported by the data before it is suitable for publication.

Reviewer 3 Comments for the Author:

Major Comments:

© 2024. Published by The Company of Biologists under the terms of the Creative Commons Attribution License (https://creativecommons.org/licenses/by/4.0/).

1. The authors propose that sites of periosteal bone resorption correspond to muscle compression (related to Figures 2 and 3). However, the authors do not perform any experimentation to confirm this hypothesis – the conclusion is based on anatomical location. As a result, the authors should carefully consider the language regarding this conclusion. Specifically, can the authors confirm that these muscles provide compression to the periosteal surfaces in question (vs. proximity with negligible mechanical force, tension, or a more complex stress state)?

As the reviewer notes, cortical bone of the hindlimb is subjected to complex stress including axial loading, tension, and compression. We measured periosteal thickness of two distinct cortical bone regions, one undergoing endo- *t*-p and the other peri-*t*-p, to determine whether the periosteum exhibits greater compaction of cells at sites of proposed compression. Periosteum of endo-*t*-p cortex was significantly thinner than that of peri-*t*-p cortex, suggesting that mechanical force is indeed applied by muscles on cortical bone exhibiting endo- *t*-p. Based on these results, which are shown in revised Fig. 3A-E, we conclude that periosteal surfaces are exposed to perpendicular muscle compression at least in developing cortical bone.

2. Using phase-contrast X-ray techniques (Fig 4), the authors nicely illustrate that cortical canals are present primarily on endo-t-p as compared to peri-t-p surfaces. However, it is not clear if the images shown are representative of the data. For example, the Fig. 4B clearly shows about 10 endo-t-p cortical canals (asterisks) and 0 peri-t-p canals. The quantification in Fig. 4C is more even between the groups, with 25 endo-t-p and 14 peri-t-p. Furthermore, it appears that only two mice were used for this experiment. It would be preferred to either perform more biological replicates or include truly representative images from both mice in the text.

Sorry for the confusion regarding endo-*t*-p versus peri-*t*-p cortical canal numbers. We now clearly indicate all canals in Fig. 4B using asterisks and arrowheads to show that the image is representative. Furthermore, we increased the sample size and now show data from 4 rather than 2 mice (revised Fig. 4C). We also added a new plot comparing peri- and endo-*t*-p cortical canal density of each mouse (revised Fig. 4D).

3. Sciatic nerve transection (SNT) is used to investigate trans-pairing and drift. However, the primary effect here seems to be related to muscle function / load on the bone rather than skeletal innervation. The authors should comment on this in the discussion as well as modify Figure 8 to include the contribution of nerves and/or muscle to their model.

As the reviewer notes, the SNT model combines muscle atrophy, denervation, disuse, and unloading. In the revision, we have now added histological analysis of the SNT model (revised Fig. 6 and supplementary Fig. S5) to demonstrate muscle atrophy and decreases in E11 expression in this model indicative of unloading and disuse effects. We also revised Fig. 8 to indicate that muscle compression induces endo-*t*-p, and that nerve injury reduces bone drift.

a. Related to this, the authors reference “inflammatory responses to sciatic nerve transection” on page 10. Since no inflammatory markers were utilized in this study, this phrase should be removed or revised.

We agree and have removed the phrase “inflammatory responses”.

4. The authors have provided a series of observations that are consistent with their proposed mechanism (Fig. 8). One perturbation (SNT) is investigated, although the mechanism does not directly test any specific aspect of the model. It may be helpful to perform a positive intervention (e.g. direct application of force to developing bone in a non-physiological direction) to directly show how these mechanisms are controlled.

Based on this comment, we performed additional experiments to directly apply force to developing bone “in a non-physiological direction” by implanting a small hex nut on calvaria under the skin. TRAP-positive areas were induced on the external surface of calvaria in response to the resulting mechanical stress.
These new results are now presented in supplementary Fig. S3.

© 2024. Published by The Company of Biologists under the terms of the Creative Commons Attribution License (https://creativecommons.org/licenses/by/4.0/).

5. The authors have extensively studied the developing mouse hindlimb, specifically the fibula. Were any other bones examined, perhaps superficially (e.g. as in Figure 1), that would illustrate the generalizability of trans-pairing in long bones?

Thank you for these comments. As shown in Fig. 1, tibia also showed *trans*- pairing, and we have also analyzed ulna and ribs. As shown in the Figure to Reviewer 3, while the interosseous border of the ulna is peri-forming, neighboring cortices are endo-*t*-p composed of periosteal TRAP-positive osteoclasts and endosteal osteoblasts (A). Rib surfaces compressed by the lung show TRAP-positive activity. We conclude that *trans*-pairing in long bones can be generalized to other bones, including the ulna and ribs. We are currently preparing our next manuscript, which generalizes *trans*-pairing beyond the mouse hindlimb.

Minor Comments:

1. If possible, information regarding the specific target or stain in each image should appear in the figure itself (e.g. in Fig 5A, the “OB OC” is helpful).

Based on this comment, we improved figures to show the specific target or stain in each image.

(Fig. 1C, D, E, F) OB and OC were added. (Fig. 3F, G) BM and OCY were added. (Fig5. D, H) OB and OC were added.

(Fig. 6G) OB, calcein, and alizarin were added. (Fig. S1) OB and OC were added.

NOTE: We have removed unpublished data that had been provided for the referees in confidence.

First revision

<u>Author response to reviewers' comments</u>

Reviewer 1 Advance summary and potential significance to field
The authors study the postnatal development of fibula and tibia and note an increase in osteoclastic resorption in the periosteum that occurs concurrently with an increase in channels connecting the periosteum and endosteum. They suggest that the new bone that forms on the endosteum is generated by preosteoblasts/osteoblasts that originated in the periosteum. if this is true, it's an important advance.

Reviewer 1 Comments for the author
The authors suggest that the new bone formation that they note on the endocortical surface comes from osteoblasts that originated from precursors at the pericondrial surface. The authors note that there are osteoblasts in canals between those surfaces that could make this possible. But they do not show that the endosteal osteoblasts originated from the periosteal precursors (as opposed to marrow precursors). Greenblatt's group published a nice paper (referenced by the authors) in which they identify periosteal precursors of osteoblasts and show that they have a gene signature that distinguishes those cells from marrow osteoblast precursors. (Shawon Debnath, Alisha R. Yallowitz, Jason McCormick, Sarfaraz Lalani, Tuo Zhang, Ren Xu, Na Li, Yifang Liu, Yeon Suk Yang, Mark Eiseman, Jae-Hyuck Shim, Meera Hameed, John H. Healey, Mathias P. Bostrom, Dan Avi Landau, and Matthew B. Greenblatt. Discovery of a periosteal stem cell mediating intramembranous bone formation. Nature. 2018 Oct; 562(7725): 133–139.) Those authors used a cre driver specific for periosteal stem cells to knock out the osterix gene and showed that this disrupted periosteal bone formation but actually increased endocortical bone formation. Certainly, in that case, the endocortical bone formation did not originate from periosteal precursors. There is still much to do to establish the fates of periosteal stem cells, but the current manuscript doesn’t mention the Greenblatt lab’s experiment that I’ve just quoted or provide any alternative direct evidence that

© 2024. Published by The Company of Biologists under the terms of the Creative Commons Attribution License (https://creativecommons.org/licenses/by/4.0/).

the endosteal osteoblasts originated in periosteal precursors. So the authors' hypothesis is intriguing and might even be right, but they need much more direct lineage data to show it.

Their demonstration of the canals between the periosteum and endosteum are the highlight of the paper and may well provide the path that the authors postulate.

>We agree that the results of Greenblatt's group may contradict our hypothesis and that more direct experimental evidence is needed to confirm that osteoblasts at the endo-forming trans-pairing (endo-t-p) cortex originate from periosteal precursors. Greenblatt's and other groups claim that periosteal cells contribute little to endocortical bone formation (Debnath et al., 2018; Jeffery et al., 2022). We feel that future studies focusing on the endo-t-p cortex separately from the peri-t-p cortex could clarify these matters.
To provide such direct evidence, we used Gli1-CreERT2 mice crossed with Ai14 reporter mice (Shi et al., 2017). Use of Gli1-CreERT2 mice allows labeling of periosteal skeletal stem cells in the diaphysis of long bones more restrictively than use of Ctsk-Cre mice (Jeffery et al., 2022). Thus, we injected 4-week-old Gli1-CreERT2 mice with tamoxifen to label those periosteal precursors, and 2 weeks later detected tdTomato-positive cells at the periosteum of the peri-t-p cortex, as expected. By contrast, tdTomato-positive cells of the endo-t-p cortex were localized in cortical canals and at the endosteum and periosteum, supporting the hypothesis that periosteal precursors migrate through cortical canals to induce endocortical bone formation. We present this new lineage-tracing data in Fig. S4 of the revision. However, we do not exclude the contribution of marrow-derived cells, and therefore have added marrow-derived preosteoblasts to the model shown in Fig. 8. We also now discuss discrepancies between the Debnath paper (2018) and this study in the revised manuscript.

Reviewer 2 Advance summary and potential significance to field
Kuroda et al present a substantial study into cell behaviors involved in bone growth. Bone is known to adapt its shape through modeling and re-modeling. Modeling is defined as "the formation of bone by osteoblasts or resorption of bone by osteoclasts on a given surface" as opposed to remodeling "in which osteoblast and osteoclast activity occur sequentially in a coupled manner on a given bone surface" ('textbook' definitions from Allen and Burr 2014, doi.org/10.1016/B978-0-12-416015-6.00004-6). Much is known about the molecular mechanisms which locally couple the activity of osteoblasts and osteoclasts. Modeling has for several decades been hypothesized to be orchestrated by osteocytes within the bone matrix (e.g. Lanyon 1993, 10.1007/BF01673415 and many subsequent papers), most importantly during functional adaptation to local mechanical strain. Mice lacking osteocytes or osteocyte-secreted proteins do not lose bone in response to mechanical disuse (e.g. Tatsumi et al Cell Metab 2007). This history is important to interpret the authors' claims including the proposal of "a fundamental mechanism underlying endo-forming transpairing, namely, triggering by periosteal osteoclasts, which then induce endocortical bone formation by enabling periosteal preosteoblasts to pass through cortical canals towards the endosteum."

Reviewer 2 Comments for the author
1. What is the evidence that endosteal formation is "triggered by periosteal osteoclasts"? None of the data presented demonstrate that periosteal osteoclasts produce a signal which is sensed by osteoblasts and increases their activity endosteally, nor is the role of osteocytes meaningfully examined.
>Accordingly, we now state in the Introduction that trans-pairing driven-bone drift occurs in an osteoclast-dependent manner based on previous analyses of osteopetrotic Tnfsf11-/- mice and mice injected with anti-RANKL antibody or osteoprotegerin (Edamoto et al., 2019; Piemontese et al., 2017). These mice exhibit impaired endocortical bone formation. Therefore, we propose that periosteal osteoclasts trigger endosteal bone formation.
>In the Results we now cite studies reporting that osteocytes act as a mechanotransducer in bone matrix (Tatsumi et al., 2007) and that E11/podoplanin is upregulated in osteocytes by mechanical loading (Zhang et al., 2006). We also performed additional experiments to detect E11 expression in fibular cortex and show that data in Fig. S5AB. That analysis confirmed that gene expression in osteocytes is altered in response to axial mechanical loading in the SNT model, as expected. However, E11 expression did not response to muscle compression, because there were no differences in osteocytic E11 expression between endo- and peri-t-p cortices in developing long bone.

© 2024. Published by The Company of Biologists under the terms of the Creative Commons Attribution License (https://creativecommons.org/licenses/by/4.0/).

2.“…enabling periosteal preosteoblasts to pass through cortical canals towards the endosteum” – what evidence is provided that pre-osteoblasts migrate through cortical channels in a periosteal to endosteal direction? The Col1a1 localisation data does not provide evidence of directional migration, particularly when closely associated with blood vessels.

>Based on this comment, we performed lineage-tracing experiments using Gli1-CreERT2 mice crossed with Ai14 reporter mice. Chasing tdTomato-positive cells after tamoxifen administration to 4-week-old mice allowed us to lineage-trace periosteal Gli1-positive osteoblast progenitors at cortical bone (Shi Y et al., 2017). Two weeks after tamoxifen injection, we detected tdTomato-positive cells at the peri-t-p cortex in the periosteum. By contrast, tdTomato-positive cells of the endo-t-p cortex localized in cortical canals and the endosteum and periosteum. These data support our hypothesis that periosteal osteoblasts travel through cortical canals towards the endosteum in the endo-t-p cortex. Thus, although the contribution of periosteal progenitors to cortical bone formation in the endo-t-p was indicated, the directional migration of Col1a1 osteoblasts remains unknown. Therefore, we have modified our model in Fig.8 to avoid excluding the possibility that osteoblast progenitors are also supplied from the bone marrow. These results are presented in Fig. S4 and Fig. 8 in the revision.

3.“Sites of periosteal bone resorption correspond to muscle compression”. This statement could just as easily read that “Sites of muscle expansion correspond to space available thanks to bone resorption”. The authors could test this proposition, at least correlatively, by determining whether muscle volumetric expansion which might compress bone precedes the onset of resorption. One might also expect greater compaction of cells on the periosteum at sites of proposed compression, which could be quantified in histological sections.

>Accordingly, we measured periosteum thickness at both endo- and peri-t-p cortices of mouse fibula at P9, P16, P23 and P30 (Fig. S2), and found that the periosteum was thinner at the endo-t-p than the peri-t-p cortex in all ages examined. These results suggest that the periosteum of endo-t-p is more compressed than that of peri-t-p and that muscle expansion induces periosteal compaction. These new data are shown in revised Figure 3E and described in the Results and Discussion.

4. The image showing in situ of Tnfsr11 is not of sufficient quality, at least in the PDF available, to exclude its expression by osteocytes. Osteocytes within the image appear to have brown dots (positive signal) in them as well.

>In response to this comment, we replaced Tnfsf11 in situ images with higher magnification images. As shown in revised Figure 3F and G, brown dots (positive signals) were most frequently observed in the cambium layer but were also detected in some osteocytes and in bone marrow.

5.“preosteoblasts weakly positive for Col1a1 were present above osteoblasts at the periosteum.” Are these different from the previously-described canopy cells, which are also associated with blood vessels (e.g. Andersen et al Am J Pathol 2009)?

>“Canopy cells” were previously reported at the endosteal surface in human bone (Andersen et al, J. Pathol, 2009). Although we observed canopy cells covering osteoclasts and osteoblasts in mouse long bone, they were Col1a1-negative. We conclude that periosteal cells weakly positive for Col1a1 differ from canopy cells.

6.The sciatic neurectomy experiment is difficult to interpret because it combines the anti-growth effect of disuse on a period of rapid growth. The reviewer’s understanding is that formation is indicated by the sequence of red (alizarin) to green (calcein) to white (osteoblast surface 2 days after calcein). It is not clear why the bottom (lateral) aspect of the neurectomised bone retained alizarin on the periosteal surface – the TRAP staining in 6D shows this is not a quiescent surface. Was it the former endosteal surface which has modelled by endosteal formation as the formely periosteal surface was resorbed (red◊green◊white)? The equivalent alizarin line in the non-neurectomised limb does in fact appear to span the periosteal to endosteal surface. A much more detailed analysis of this data, likely including quantification of inter-label distance for bone formation rates and multi-site comparisons to help interpret drift direction, is needed.

© 2024. Published by The Company of Biologists under the terms of the Creative Commons Attribution License (https://creativecommons.org/licenses/by/4.0/).

>We agree with the reviewer's interpretation of the histological analysis and now describe double-labeling and osteocalcin immunostaining in greater detail. Moreover, we now analyze paraffin sections (using both H&E and TRAP staining) in addition to frozen sections to identify drift directions.

7.Sciatic neurectomy decreases muscle volume, yet osteoclast surface per bone surface (inferred from TRAP staining) is not decreased. This argues against the assertion that "osteoclastic bone resorption surfaces are established in response to muscle compression".

>Inspired by the reviewer, we now detect weaker TRAP activity at the periosteum of the anterior cortex after SNT corresponding to observed muscle atrophy. We described this in Results and Discussion in revision.

8.Regarding the larger trans-cortical canals following neurectomy: can the authors determine whether disuse stopped in-filling of canals which were large at the start of the experiment or caused expansion of canals which were smaller at the start (e.g. by comparison with canals in the tibiae of mice at P16)?

>We agree that it would be interesting to determine whether the larger cortical canals were initially large, or whether small canals expand to become large. Analysis of a disuse muscle atrophy model with tail-suspension experiments is one approach, and indeed we have submitted an animal study protocol to the Institutional Animal Care and Use Committee (IACIC) to conduct such analyses in the future. However, here, to address the reviewer's question, we performed additional bone labeling experiments on fibulae with or without SNT and conducted lightsheet microscopy analysis (Fig. S6). In SNT-treated fibula, intracortical labeling was decreased compared with control fibula. These data suggest that the increase in cortical canal number was due to stopping the in-filling of canals.

9. In Figure 4C, the number of mice analysed is not clear. The individual mouse, not multiple canals within the same mouse, should be used as the unit of measure.

>Accordingly, we increased the number of mice from 2 to 4 and now indicate this in the Figure 4C legend. We also now show the number of canals per bone surface area (canal density) of individual mice in Figure 4D in the revision.

10. Were male or female mice used for the experiments?

>We now provide the number of male and female mice analyzed (underlined) in each figure legend.

Minor:
11.The concept of trans-pairing, while interesting, needs to be put in context of what is known about modeling and remodeling in the introduction and discussion, particularly in species without Haversian canals.

>Thank you for this comment. Accordingly, we added an explanation of modeling and remodeling to the revised Introduction and Discussion.

Reviewer 3 Advance summary and potential significance to field
In this study, the authors have examined the coordinated activity of periosteal and endosteal osteoblasts and osteoclasts in developing long bones. Through a series of in vivo experiments, the authors posit that osteoclast activation at the periosteal surface, potentially due to muscle and/or nerve-induced signals, results in the migration of preosteoblasts through cortical canals to the endocortical surface to produce new bone tissue. The experiments are well described, the manuscript itself is well-written, and the figures are clear and compelling. However, more effort needs to be dedicated to ensuring that the conclusions are supported by the data before it is suitable for publication.

Reviewer 3 Comments for the author
Major Comments:

© 2024. Published by The Company of Biologists under the terms of the Creative Commons Attribution License (https://creativecommons.org/licenses/by/4.0/).

1.The authors propose that sites of periosteal bone resorption correspond to muscle compression (related to Figures 2 and 3). However, the authors do not perform any experimentation to confirm this hypothesis – the conclusion is based on anatomical location. As a result, the authors should carefully consider the language regarding this conclusion. Specifically, can the authors confirm that these muscles provide compression to the periosteal surfaces in question (vs. proximity with negligible mechanical force, tension, or a more complex stress state)?

>As the reviewer notes, cortical bone of the hindlimb is subjected to complex stress including axial loading, tension, and compression. We measured periosteal thickness of two distinct cortical bone regions, one undergoing endo-t-p and the other peri-t-p, to determine whether the periosteum exhibits greater compaction of cells at sites of proposed compression. Periosteum of endo-t-p cortex was significantly thinner than that of peri-t-p cortex, suggesting that mechanical force is indeed applied by muscles on cortical bone exhibiting endo-t-p. Based on these results, which are shown in revised Fig. 3A-E, we conclude that periosteal surfaces are exposed to perpendicular muscle compression at least in developing cortical bone.

2.Using phase-contrast X-ray techniques (Fig 4), the authors nicely illustrate that cortical canals are present primarily on endo-t-p as compared to peri-t-p surfaces. However, it is not clear if the images shown are representative of the data. For example, the Fig. 4B clearly shows about 10 endo-t-p cortical canals (asterisks) and 0 peri-t-p canals. The quantification in Fig. 4C is more even between the groups, with 25 endo-t-p and 14 peri-t-p. Furthermore, it appears that only two mice were used for this experiment. It would be preferred to either perform more biological replicates or include truly representative images from both mice in the text.

>Sorry for the confusion regarding endo-t-p versus peri-t-p cortical canal numbers. We now clearly indicate all canals in Fig. 4B using asterisks and arrowheads to show that the image is representative. Furthermore, we increased the sample size and now show data from 4 rather than 2 mice (revised Fig. 4C). We also added a new plot comparing peri- and endo-t-p cortical canal density of each mouse (revised Fig. 4D).

3. Sciatic nerve transection (SNT) is used to investigate trans-pairing and drift. However, the primary effect here seems to be related to muscle function / load on the bone rather than skeletal innervation. The authors should comment on this in the discussion as well as modify Figure 8 to include the contribution of nerves and/or muscle to their model.

>As the reviewer notes, the SNT model combines muscle atrophy, denervation, disuse, and unloading. In the revision, we have now added histological analysis of the SNT model (revised Fig. 6 and supplementary Fig. S5) to demonstrate muscle atrophy and decreases in E11 expression in this model indicative of unloading and disuse effects. We also revised Fig. 8 to indicate that muscle compression induces endo-t-p, and that nerve injury reduces bone drift.

a. Related to this, the authors reference “inflammatory responses to sciatic nerve transection” on page 10. Since no inflammatory markers were utilized in this study, this phrase should be removed or revised.

>We agree and have removed the phrase “inflammatory responses”.

4. The authors have provided a series of observations that are consistent with their proposed mechanism (Fig. 8). One perturbation (SNT) is investigated, although the mechanism does not directly test any specific aspect of the model. It may be helpful to perform a positive intervention (e.g. direct application of force to developing bone in a non-physiological direction) to directly show how these mechanisms are controlled.

>Based on this comment, we performed additional experiments to directly apply force to developing bone “in a non-physiological direction” by implanting a small hex nut on calvaria under the skin. TRAP-positive areas were induced on the external surface of calvaria in response to the resulting mechanical stress. These new results are now presented in supplementary Fig. S3.

© 2024. Published by The Company of Biologists under the terms of the Creative Commons Attribution License (https://creativecommons.org/licenses/by/4.0/).

5.The authors have extensively studied the developing mouse hindlimb, specifically the fibula. Were any other bones examined, perhaps superficially (e.g. as in Figure 1), that would illustrate the generalizability of trans-pairing in long bones?

>Thank you for these comments. As shown in Fig. 1, tibia also showed trans-pairing, and we have also analyzed ulna and ribs. As shown in the Figure to Reviewer 3, while the interosseous border of the ulna is peri-forming, neighboring cortices are endo-t-p composed of periosteal TRAP-positive osteoclasts and endosteal osteoblasts (A). Rib surfaces compressed by the lung show TRAP-positive activity. We conclude that trans-pairing in long bones can be generalized to other bones, including the ulna and ribs. We are currently preparing our next manuscript, which generalizes trans-pairing beyond the mouse hindlimb.

Minor Comments:

1.If possible, information regarding the specific target or stain in each image should appear in the figure itself (e.g. in Fig 5A, the “OB OC” is helpful).

>Based on this comment, we improved figures to show the specific target or stain in each image.
(Fig. 1C, D, E, F) OB and OC were added.
(Fig. 3F, G) BM and OCY were added.
(Fig5. D, H) OB and OC were added.
(Fig. 6G) OB, calcein, and alizarin were added.
(Fig. S1) OB and OC were added.

<u>Second decision letter</u>

MS ID#: DEVELOP/2023/202194

MS TITLE: Developing long bones respond to surrounding tissues by *trans*-pairing of periosteal osteoclasts and endocortical osteoblasts

AUTHORS: Yukiko Kuroda, Masaki Yoda, Katsuhiro Kawaai, Motoharu Tatenuma, Toshihide Mizoguchi, Yanlin Wu, Hidekazu Takano, Atsushi Momose, and Koichi Matsuo

I have now received all the referees' reports on the above manuscript, and have reached a decision. The referees' comments are appended below, or you can access them online: please go to BenchPress and click on the 'Manuscripts with Decisions' queue in the Author Area.

As you will see, two of referees have expressed continuous concerns and recommend further revision of your manuscript before we can consider publication. If you are able to revise the manuscript along the lines suggested, which may involve further experiments, I will be happy to receive a revised version of the manuscript. Your revised paper will be re-reviewed by one or more of the original referees, and acceptance of your manuscript will depend on your addressing satisfactorily the reviewers' major concerns. Please also note that Development will normally permit only one round of major revision. If it would be helpful, you are welcome to contact us to discuss your revision in greater detail. Please send us a point-by-point response indicating your plans for addressing the referees’ comments, and we will look over this and provide further guidance.

Please attend to all of the reviewers' comments and ensure that you clearly highlight all changes made in the revised manuscript. Please avoid using 'Tracked changes' in Word files as these are lost in PDF conversion. I should be grateful if you would also provide a point-by-point response detailing how you have dealt with the points raised by the reviewers in the 'Response to Reviewers' box. If you do not agree with any of their criticisms or suggestions please explain clearly why this is so.

© 2024. Published by The Company of Biologists under the terms of the Creative Commons Attribution License (https://creativecommons.org/licenses/by/4.0/).

Reviewer 1

Advance summary and potential significance to field

See my previous review

Comments for the author

The authors have improved their manuscript substantially. With regard to my request for lineage tracing, they performed the experiment shown in Figure S4.
Gli1-CreERT2; Ai14 (tomato reporter) mice were given tamoxifen at 4 weeks of age and examined at one subsequent time point (6 weeks of age). At that time the authors see tomato+ cells in the periosteum and in cortical canals and endosteal cortex. Guessing that tdTomato is only expressed in the periosteum at 4 weeks of age, the authors conclude that some of the tdTomato-expressing cells move, two weeks later into canals and then to the endocortex. The authors may be right. They refer to the study of Shi et al that showed that at early times after tamoxifen, tdTomato expression occurs in the perichondrium (and other sites in bone). But Shi et al did not look at the fibula at four weeks of age, so I can't be sure that the fibula doesn't' express tdTomato at four weeks of age. If it did, then the whole idea of tracing lineage wouldn't work. So this is a nice study but is missing an early time point that shows tdTomato in the periosteum but nowhere else. Then, after tamoxifen goes away, the tdTomato+ cells away from the periosteum must have descended from the periosteal cells (or be those cells after migration). But without the early time point, the current time point cannot be interpreted. (It is not enough to say that Shi sees only periosteal cells at an early time point.)

Reviewer 2

Advance summary and potential significance to field

The authors have made considerable changes to the manuscript and performed new experiments which do not reassure this reviewer of the validity of their pre-osteoblasts migrate through vascular channels to couple bone formation and resorption across the cortex of mouse long bones.

Comments for the author

Major comments:
1. The new 'small hex nut' experiment is not described in the methods.
Irrespective, it is unlikely to be conclusive. Implantation of a foreign body over the calvarium is guaranteed to cause an inflammatory response, which is well known to increase resorption. The claim that this tests "whether mechanical compression induces osteoclastic bone resorption" needs to be removed, and in any case it is well known that static compression would promote bone loss (e.g. Lanyon and Rubin J Biomech 1984 – quantitative and controlled analysis of static versus dynamic loads in long bones).
2. The new lineage tracing experiment is described as showing "tdTomato-positive cells of the endo-t-p cortex were localized in cortical canals and at the endosteum and periosteum, supporting the hypothesis that periosteal precursors migrate through cortical canals to induce endocortical bone formation." It could have addressed my initial concern if the authors had quantified tdTom lineage-traced cells at different distances into the cortical canals on different time points. The incredibly sparse labelling of tdTom lineage-traced spots and visualization at a single timepoint makes this experiment impossible to interpret. Some of the spots indicated are not convincingly cells at the magnification and high contrast shown (e.g. spot shown by the arrowhead in the bottom panel of Supp Fig 4B, which is less convincing than the spot in the muscle at the top right of panel A). The methods of this experiment are not provided in sufficient detail (dose of tamoxifen? Replicates?), and without quantification of sufficient lineage-traced cells at different timepoints does not support the conclusion.
3. I apologise if my previous comment about periosteal thickness led the authors to compare the thickness of an active, cuboidal osteoblasts on a bone-forming periosteum versus a quiescent surface, unsurprisingly finding the latter is thinner. The conclusion that "the periosteum of the

© 2024. Published by The Company of Biologists under the terms of the Creative Commons Attribution License (https://creativecommons.org/licenses/by/4.0/).

endo-t-p cortex is more compressed than that of peri-t-p cortex" cannot be made from this comparison.
More relevant comparisons would have been between two quiescent surfaces with and without abutting muscle, or on the same site with and without sciatic neurectomy (reduced muscle).
4. "In SNT-treated fibula, E11 expression was lower than in controls, and E11 protein in osteocyte dendrites was barely detectable (Fig. S5A, B, SNT (R)),
suggesting that muscle compression and/or axial mechanical loading is indeed reduced in the SNT model." Several previous studies clearly show that osteocyte mechano-responses following sciatic neurectomy are rescued by axial loading which does not rescue muscle mass. The suggestion that the increase in E11 could be due to changes in muscle compression are inconsistent with that previous literature and should be removed.

Reviewer 3

Advance summary and potential significance to field

This manuscript provides new understanding of how developing bone changes in response to mechanical stress or nerve injury as well as the crosstalk between endosteal and periosteal compartments.

Comments for the author

I have no additional comments. This manuscript is appropriate for publication.

Second revision

Author response to reviewers' comments

Reviewer 1 Comments for the author
The authors have improved their manuscript substantially. With regard to my request for lineage tracing, they performed the experiment shown in Figure S4. Gli1-CreERT2; Ai14 (tomato reporter) mice were given tamoxifen at 4 weeks of age and examined at one subsequent time point (6 weeks of age). At that time the authors see tomato+ cells in the periosteum and in cortical canals and endosteal cortex. Guessing that tdTomato is only expressed in the periosteum at 4 weeks of age, the authors conclude that some of the tdTomato-expressing cells move, two weeks later into canals and then to the endocortex. The authors may be right. They refer to the study of Shi et al that showed that at early times after tamoxifen, tdTomato expression occurs in the perichondrium (and other sites in bone). But Shi et al did not look at the fibula at four weeks of age, so I can't be sure that the fibula doesn't express tdTomato at four weeks of age. If it did, then the whole idea of tracing lineage wouldn't work. So this is a nice study but is missing an early time point that shows tdTomato in the periosteum but nowhere else. Then, after tamoxifen goes away, the tdTomato+ cells away from the periosteum must have descended from the periosteal cells (or be those cells after migration). But without the early time point, the current time point cannot be interpreted. (It is not enough to say that Shi sees only periosteal cells at an early time point.)

Responses to Reviewer 1:
Thank you for this critical comment. We agree that analyses at early and late time points are necessary to interpret the direction of cell migration using lineage tracing experiment. We have analyzed the fibular diaphysis of Gli1-CreERT2; Ai14 mice immediately after tamoxifen administration. As shown in revised Fig. 8B, tdTomato-positive cells were localized in the periosteum, but not in the endosteum, 24 h after tamoxifen administration. From these results, we conclude that tdTomato-positive cells were localized in the periosteum of the fibular diaphysis immediately after tamoxifen injection and that two weeks later some of the tdTomato-expressing cells migrate into cortical canals and the endosteum at the endo-t-p cortex.

Reviewer 2 Comments for the author

© 2024. Published by The Company of Biologists under the terms of the Creative Commons Attribution License (https://creativecommons.org/licenses/by/4.0/).

Major comments:
1.The new ‘small hex nut’ experiment is not described in the methods. Irrespective, it is unlikely to be conclusive. Implantation of a foreign body over the calvarium is guaranteed to cause an inflammatory response, which is well known to increase resorption. The claim that this tests “whether mechanical compression induces osteoclastic bone resorption” needs to be removed, and in any case it is well known that static compression would promote bone loss (e.g. Lanyon and Rubin J Biomech 1984 – quantitative and controlled analysis of static versus dynamic loads in long bones).

Responses to Reviewer 2-1:
The method of hex nut implantation was described after the figures in Supplementary information. This experiment was performed in response to the reviewer 3’s previous comment 4: “It may be helpful to perform a positive intervention (e.g. direct application of force to developing bone in a non-physiological direction) to directly show how these mechanisms are controlled”. We tested mechanical compression perpendicular to the cortical surface by applying non-physiological loads on mouse calvaria under the nut. In this regard, we think Lanyon and Rubin J Biomech 1984 paper describes a very different situation where mechanical compression was indirectly and longitudinally applied to the cortex of turkey long bone. On the other hand, we think that you correctly pointed out that inflammation due to the foreign body implantation or friction upon nut movement could induce bone resorption. Therefore, we have added possible inflammation-induced bone resorption caused by a foreign body implantation in the revised manuscript as follows.
>P6, Results: “The data suggest that mechanical compression applied perpendicular to the cortical surface may induce bone resorption. Nevertheless, the potential for inflammation-induced bone resorption cannot be ruled out.”

2.The new lineage tracing experiment is described as showing “tdTomato-positive cells of the endo-t-p cortex were localized in cortical canals and at the endosteum and periosteum, supporting the hypothesis that periosteal precursors migrate through cortical canals to induce endocortical bone formation.” It could have addressed my initial concern if the authors had quantified tdTom lineage-traced cells at different distances into the cortical canals on different time points. The incredibly sparse labelling of tdTom lineage-traced spots and visualization at a single timepoint makes this experiment impossible to interpret. Some of the spots indicated are not convincingly cells at the magnification and high contrast shown (e.g. spot shown by the arrowhead in the bottom panel of Supp Fig 4B, which is less convincing
than the spot in the muscle at the top right of panel A). The methods of this experiment are not provided in sufficient detail (dose of tamoxifen? Replicates?), and without quantification of sufficient lineage-traced cells at different timepoints does not support the conclusion.

Responses to Reviewer 2-2:
We agree that analyses at different timepoints especially early and late timepoints by observing more “lineage-traced cells” are necessary to support the conclusion. We have analyzed the fibular diaphysis of Gli1-CreERT2; Ai14 mice immediately after tamoxifen administration. As shown in revised Fig. 8B, tdTomato-positive cells were found in the periosteum, but not in the endosteum, 24 h after tamoxifen. From these results, we concluded that tdTomato-positive cells were localized in the periosteum of the fibular diaphysis immediately after tamoxifen injection and that some of the tdTomato-expressing cells were migrated into cortical canals and the endosteum at the endo-t-p cortex in two weeks. To ensure the detection of tdTomato-positive cells, we prepared thicker sections from samples at two time points (24 h and 2 weeks after tamoxifen injection) and performed immunostaining with anti-tdTomato antibody. By improving methods, we now show more clearly the presence of tdTomato-positive cells in the canal and the endosteum (revised Fig. 8B-D) than in the previous manuscript.
The methods were described in the Supplementary Information in the previous version, but in the revised version, we transferred the results of lineage tracing experiments together with the methods to the main manuscript (revised Fig. 8).

3.I apologise if my previous comment about periosteal thickness led the authors to compare the thickness of an active, cuboidal osteoblasts on a bone-forming periosteum versus a quiescent surface, unsurprisingly finding the latter is thinner. The conclusion that “the periosteum of the endo-t-p cortex is more compressed than that of peri-t-p cortex” cannot be made from this comparison. More relevant comparisons would have been between two quiescent surfaces with and

© 2024. Published by The Company of Biologists under the terms of the Creative Commons Attribution License (https://creativecommons.org/licenses/by/4.0/).

without abutting muscle, or on the same site with and without sciatic neurectomy (reduced muscle).

Responses to Reviewer 2-3:
We understood your points and compared the periosteal thickness of the anterior cortices with and without sciatic neurectomy. The anterior cortex of the control fibula is in contact with muscles and undergoes endo-t-p. As shown in the revised Figure 6D, SNT-treatment increased the periosteum thickness of the anterior cortex. This result clearly indicates that muscles perpendicularly compress the anterior cortex of the fibula under the physiological development. We show the new data in the revised Figure 6D, and described as follows.
>P9, Results: “Moreover, muscle atrophy by SNT-treatment increased the thickness of the anterior periosteum, indicating that the SNT-treated fibula has reduced perpendicular compression by the surrounding muscle (Fig. 6D).”

4.“In SNT-treated fibula, E11 expression was lower than in controls, and E11 protein in osteocyte dendrites was barely detectable (Fig. S5A, B, SNT (R)), suggesting that muscle compression and/or axial mechanical loading is indeed reduced in the SNT model.” Several previous studies clearly show that osteocyte mechano-responses following sciatic neurectomy are rescued by axial loading, which does not rescue muscle mass. The suggestion that the increase in E11 could be due to changes in muscle compression are inconsistent with that previous literature and should be removed.

Responses to Reviewer 2-4:
Thank you for the suggestion. We agree with your point and delete “muscle compression and/or” from this sentence in P9, Results.

Third decision letter

MS ID#: DEVELOP/2023/202194

MS TITLE: Developing long bones respond to surrounding tissues by *trans*-pairing of periosteal osteoclasts and endocortical osteoblasts

AUTHORS: Yukiko Kuroda, Masaki Yoda, Katsuhiro Kawaai, Motoharu Tatenuma, Toshihide Mizoguchi, Shinichirou Ito, Masataka Kasahara, Yanlin Wu, Hidekazu Takano, Atsushi Momose, and Koichi Matsuo

I am happy to tell you that your manuscript has been accepted for publication in Development, pending our standard publication integrity checks.

Reviewer 1

Advance summary and potential significance to field

See my comments in previous reviews. The demonstration of movement of cells from the periosteum to the endosteum is novel and important.

Comments for the author

I think that the changes made now by the authors effectively address the concerns I raised.

© 2024. Published by The Company of Biologists under the terms of the Creative Commons Attribution License (https://creativecommons.org/licenses/by/4.0/).
